# Facile Synthesis of Polysubstituted Pyridines via Metal-Free [3+3] Annulation Between Enamines and β,β-Dichloromethyl Peroxides

**DOI:** 10.3390/ijms26157105

**Published:** 2025-07-23

**Authors:** Yangyang Ma, Hua Zhang, Zhonghao Zhou, Chenyang Yang, Wenxiao Chang, Mohan Li, Yapei Zheng, Weizhuang Zhang, Huan Yue, Changdong Chen, Ming La, Yongjun Han

**Affiliations:** 1 School of Chemical & Environmental Engineering, Pingdingshan University, Pingdingshan 467000, China; mayang66789@163.com (Y.M.); 18837989818@163.com (C.Y.); 17746328684@163.com (W.C.); lmh209449@163.com (M.L.); 19138110149@163.com (Y.Z.); yuehuan66@outlook.com (H.Y.); lmccd5613@163.com (C.C.); laming82@126.com (M.L.); 2 College of Medicine, Pingdingshan University, Pingdingshan 467000, China; zhanghua6112@126.com; 3 School of Material Science and Engineering, Dalian Jiaotong University, Dalian 116028, China; 4 Malaga School of Engineering, Pingdingshan University, Pingdingshan 467000, China; 231082027@e.pdsu.edu.cn

**Keywords:** β,β-dichloromethyl peroxides, [3+3] annulation, polysubstituted pyridines

## Abstract

Our work introduces a facile and efficient metal-free [3+3] annulation approach for the synthesis of polysubstituted pyridines via the reaction between β-enaminonitriles and β,β-dichloromethyl peroxides. This strategy operates under mild conditions, demonstrating broad substrate scope and excellent functional group tolerance. Mechanistic investigations suggest that the reaction proceeds through a Kornblum–De La Mare rearrangement followed by S_N_V-type C-Cl bond cleavage and intramolecular cyclization/condensation. By circumventing the need for transition metal catalysts or radical initiators, our method offers practical utility in organic synthesis and provides a new avenue for the rapid construction of complex pyridine scaffolds.

## 1. Introduction

**Scheme 1:**
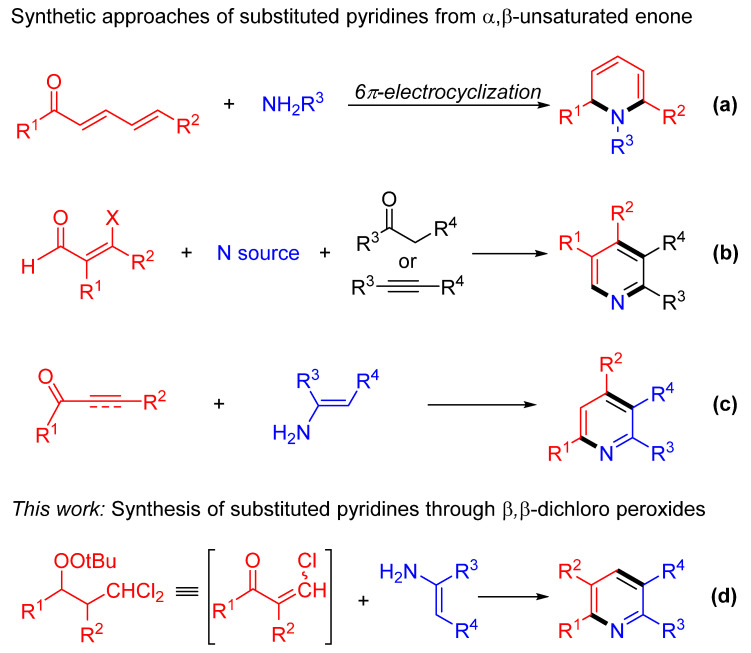
Synthesis of polysubstituted pyridines from α,β-unsaturated enyne ketones.

**Figure 1:**
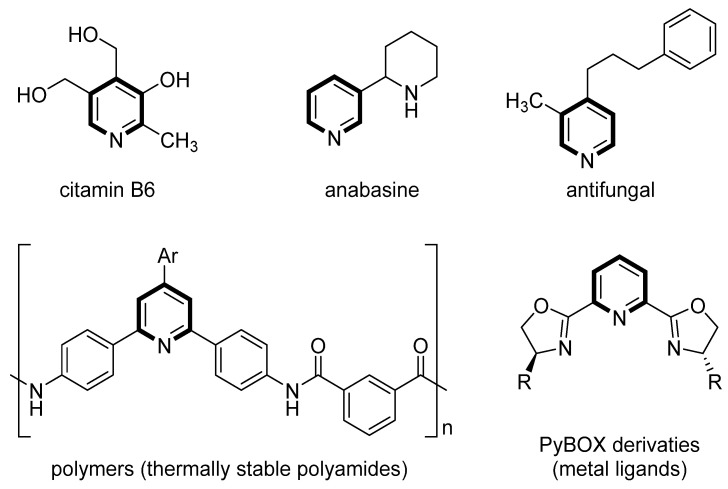
Representative compounds containing a pyridine substructure.

The pyridine heterocyclic framework demonstrates exceptional functional versatility across pharmaceutical, agrochemical, and materials science applications (Figure 1), which arises from its synergistic interplay of electronic delocalization and nitrogen-centered acidity/basicity [1,2,3,4]. This structural feature enables nitrogen to mediate hydrogen bonding, metal coordination, and redox-active transformations and other key mechanistic determinants that underpin its biological activity and technological utility [5]. Consequently, the development of efficient synthetic methodologies for accessing polysubstituted pyridines remains a cornerstone of organic chemistry. In general, there are mainly three intermolecular annulation strategies to assemble various polysubstituted pyridines [1,2,3,4] (Scheme 1): (1) Cyclocondensation of 2,4-dienone derivatives with amines, which activates the dienone’s conjugated π-system with amine nitrogen to form pyridines, though regiocontrol demands precise reaction conditions (**path a**) [6,7,8,9]. Although this method represents a classical approach for synthesizing pyridines, it is often limited by poor functional group compatibility and demanding reaction conditions. (2) [3+2+1] Annulation of β-substituted-α,β-unsaturated ketones/aldehydes with nitrogen sources and secondary carbonyl compounds, enabling sequential nucleophilic/electrophilic additions but often requiring stoichiometric additives or elevated temperatures (**path b**) [10,11,12,13,14,15,16]. [3+2+1] Annulation offers modular assembly but typically requires transition metals or radical initiators, which may complicate scalability. And (3) [3+3] annulation of α,β-unsaturated alkynes with enamines, leveraging alkyne triple bonds and enamine enolates for cycloaddition, albeit frequently relying on transition metal catalysts or radical initiators, limiting scalability (**path c**) [17,18,19,20,21,22,23,24,25,26].

Organic peroxides have attracted widespread attention due to their unique bioactive properties and oxidative capabilities in the fields of biochemistry, pharmaceuticals, and food chemistry [27,28]. Meanwhile, owing to their distinctive reactivity, organic peroxides have been further utilized as key intermediates in organic synthesis, playing a crucial role in enabling various transformations, such as the synthesis of ketones, alcohols, and epoxides [29,30,31]. β,β-Dihaloperoxides as a special peroxide compound exhibit notable advantages including facile synthetic accessibility [32,33,34,35], superior chemical stability [30], and exceptional chemoselectivity [36,37,38,39,40,41] in reactive processes. These attributes render them valuable synthetic building blocks that are extensively utilized in diverse organic transformations and strategic bond-forming reactions [32,33,34,35,36,37,38,39,40,41]. In this context, our work introduces a facile, metal-free [3+3] annulation strategy for the synthesis of polysubstituted pyridines via the reaction of β-enaminonitriles with β,β-dichloromethyl peroxides (Scheme 1d). This approach circumvents the need for transition metals, operates under mild conditions, and demonstrates broad substrate tolerance, offering a practical alternative to existing methods.

## 2. Results and Discussions

We first examined the reaction between β,β-dichloromethyl peroxide (**1a**, **Table S1**) and 3-aminocrotononitrile (**2a**) in the presence of KOH as the base and MeCN as the solvent; the desired pyridine **3a** was obtained in 55% yield, while 31% of the β,β-dichloromethyl peroxide remained unreacted (entry 1, Table 1). Subsequently, in screening with organic bases such as NEt_3_ and DIPEA (entry 2 and 3), no target product was observed under these conditions, with the starting materials remaining largely unreacted. Replacing organic bases with inorganic such as K_3_PO_4_ and Cs_2_CO_3_ (entry 4 and 5) similarly failed to effectively promote the reaction, whereas NaOH exhibited a reaction efficiency comparable to that of KOH (entry 6). Various solvents were tested in the reaction, including DMF, DMSO, THF, MeOH, EA, and DCM, among which highly polar solvents DMSO and DMF exhibited superior reactivity. This likely stems from the enhanced solubility of the base in high-polarity solvents. When KOH was employed as the base and DMSO as the solvent, elevating the reaction temperature afforded the target product **3a** in 73% yield.

With the optimized conditions established, the scopes of substrates **1** and **2** were investigated (Table 2). Representative results are summarized in Table 2. β,β-dichloromethyl peroxides **1** bearing a variety of functional groups (R = aryl), including both the electron-donating (CH_3_, tBu, and OCH_3_) and electron-withdrawing (Cl, Br, and F) groups, reacted smoothly with 3-aminocrotononitrile **2a**. The target products **3a**–**3k** were obtained in moderate to good yields regardless of the substitution position (ortho-, meta-, or para-) on the benzene ring. As expected, an additional π-extended system such as naphthalene (**3l**) was also applicable to this transformation. Similarly, the 2,3,5,6- tetrasubstituted product **3m** was obtained in 63% yield. When the methyl of **2a** is replaced with a phenyl (**2b**), the target product **3n** can be obtained with 72%. Moreover, substitution of the cyanomethyl group in **2a** with other electron-withdrawing substituents, such as ketone (**2c**) or ester (**2d**), yielded the desired products **3o** (64%) and **3p** (67%), respectively.

We further performed 1.0 mmol scale experiments under the modified reaction conditions and the desired product **3a** was obtained in 63% yield (Table 2). And the synthesized 3-cyanopyridine can be utilized in further chemical transformations, such as hydrolysis and Hofmann rearrangement, which have been demonstrated by Dai and colleagues [42].

**Scheme 2:**
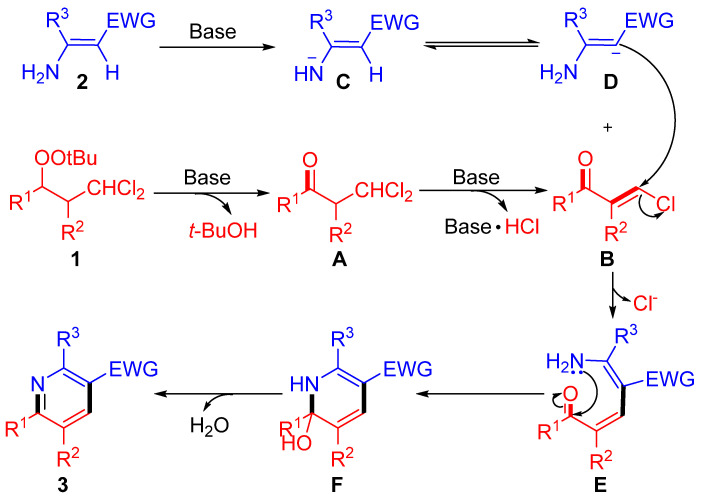
Proposed reaction mechanisms.

On the basis of the results presented above and reports in the literature, a plausible mechanism for this [3+3] annulation reaction was proposed (Scheme 2) [37,38,39]. The base-mediated Kornblum–De La Mare rearrangement of peroxide **1** and the further elimination of one molecule of HCl affords the key β-chloro enone **B**. Concurrently, β-enamine bearing electron-withdrawing groups (**2**) undergoes base-induced deprotonation, resulting in the generation of a resonance-stabilized carbon anion **D**. Then, S_N_V-type C−Cl bond cleavage of **B** with the carbanion of **D** preferentially and readily produces the intermediate E. Subsequent intramolecular cyclocondensation via **F** leads to the formation of the desired product **3**.

## 3. Materials and Methods

### 3.1. General Information

^1^H NMR spectra were recorded on a Bruker (Massachusetts, USA) 400/600 MHz spectrometer and the chemical shifts were reported in parts per million (*δ*) relative to internal standard TMS (0 ppm) for CDCl_3_. The peak patterns are indicated as follows: s, singlet; d, doublet; dd, doublet of doublet; t, triplet; m, multiplet; q, quartet. The coupling constants, *J*, are reported in Hertz (Hz). ^13^C NMR spectra were obtained at 100 MHz and 150 MHz and referenced to the internal solvent signals (the central peak is 77.0 ppm in CDCl_3_) and to the internal solvent signals (the central peak is 39.9 ppm in DMSO). CDCl_3_ and DMSO were used together as the NMR solvent. APEX II (Bruker Inc., Karlsruhe, Germany) was used for ESI-MS and EI-MS. IR spectra were recorded by means of a Bruker Tensor 27 infrared spectrometer. Flash column chromatography was performed over silica gel 200–300. All reagents were weighed and handled in air at room temperature. All chemical reagents were purchased from Alfa (Shanghai, China), Acros (Shanghai, China), Aldrich (Shanghai, China), TCI (Shanghai, China), Energy (Shanghai, China), and J&K (Shanghai, China) and used without further purification.

### 3.2. General Procedures for the Synthesis of β,β-Dichloromethyl Peroxides 1

A 25 mL round-bottom flask equipped with a magnetic stir bar was charged with CuI (1.9 mg, 1.0 mol%), alkene (1.0 mmol), CHCl_3_ (2.5 mL), acetone (2.5 mL), DIPEA (870 uL, 5.0 eq.), and 70% aqueous TBHP (650 μL, 5.0 eq.) in the order listed. The flask was placed in a water bath and allowed to stir at room temperature for 2–5 h. The resulting mixture and the solvent were evaporated under vacuum. The residue was purified by flash column chromatography on silica gel (eluent: ethyl acetate/petroleum ether) to give the peroxides **1a**–**1m** (isolated yields: 83–91%) [24].

### 3.3. General Procedures for the Synthesis of Pyridines 3

To a Schlenk tube were added KOH (0.6 mmol), **2** (0.4 mmol), dichloromethyl-peroxides (**1a**–**1r**) (0.2 mmol), and DMSO (2.0 mL) at room temperature and the resulting solution was stirred for 5 h. The resulting mixture and the solvent were evaporated under vacuum. The residue was purified by flash column chromatography on silica gel (eluent: ethyl acetate/petroleum ether) to give the polysubstituted pyridines (**3a**–**3p**).

*2-Methyl-6-(p-tolyl)nicotinonitrile* (**3a**). (32.4 mg, 78%). Isolated by flash column chromatography (eluent: ethyl acetate/petroleum ether = 1:20, R*_f_* = 0.3); white solid; ^1^H NMR (400 MHz, CDCl_3_) *δ* 7.93 (d, *J* = 8.1 Hz, 2H), 7.88 (d, *J* = 8.2 Hz, 1H), 7.61 (d, *J* = 8.2 Hz, 1H), 7.29 (d, *J* = 8.0 Hz, 2H), 2.82 (s, 3H), 2.42 (s, 3H); ^13^C NMR (100 MHz, CDCl_3_) δ 161.5, 159.7, 140.7, 140.5, 134.9, 129.7, 127.3, 117.5, 116.9, 106.5, 23.9, 21.4; HRMS (ESI) calcd for C_14_H_13_N_2_^+^ [M+H^+^]: 209.1073; found: 209.1066.

*6-(4-(Tert-butyl)phenyl)-2-methylnicotinonitrile* (**3b**). (36.5 mg, 73%). Isolated by flash column chromatography (eluent: ethyl acetate/petroleum ether = 1:20, R*_f_* = 0.3); white solid; ^1^H NMR (400 MHz, CDCl_3_) δ 7.96 (d, *J* = 8.6 Hz, 2H), 7.90 (d, *J* = 8.2 Hz, 1H), 7.62 (d, *J* = 8.2 Hz, 1H), 7.51 (d, *J* = 8.6 Hz, 2H), 2.83 (s, 3H), 1.36 (s, 9H); ^13^C NMR (100 MHz, CDCl_3_) *δ* 161.5, 159.8, 153.8, 140.5, 135.0, 127.2, 126.0, 117.5, 117.1, 106.5, 34.9, 31.2, 23.9; HRMS (ESI) calcd for C_17_H_19_N_2_^+^ [M+H^+^]: 251.1543; found: 251.1534.

*6-(4-Methoxyphenyl)-2-methylnicotinonitrile* (**3c**). (34.9 mg, 71%). Isolated by flash column chromatography (eluent: ethyl acetate/petroleum ether = 1:20, R*_f_* = 0.3); white solid; ^1^H NMR (400 MHz, CDCl_3_) *δ* 8.03–8.01 (m, 2H), 7.87 (d, *J* = 8.2 Hz, 1H), 7.58 (d, *J* = 8.2 Hz, 1H), 7.02–7.00 (m, 2H), 3.87 (s, 3H), 2.81 (s, 3H); ^13^C NMR (100 MHz, CDCl_3_) *δ* 161.6, 161.5, 159.3, 140.5, 130.2, 128.9, 117.6, 116.4, 114.4, 106.0, 55.4, 23.9; HRMS (ESI) calcd for C_14_H_13_N_2_O^+^ [M+H^+^]: 247.0842; found: 247.0832.

*2-Methyl-6-phenylnicotinonitrile* (**3d**). (27.5 mg, 78%). Isolated by flash column chromatography (eluent: ethyl acetate/petroleum ether = 1:20, R*_f_* = 0.3); white solid; ^1^H NMR (400 MHz, CDCl_3_) *δ* 8.05–8.03 (m, 2H), 7.93 (d, *J* = 8.2 Hz, 1H), 7.65 (d, *J* = 8.2 Hz, 1H), 7.53–7.47 (m, 3H), 2.84 (s, 3H); ^13^C NMR (100 MHz, CDCl_3_) *δ* 161.6, 159.8, 140.7, 137.7, 130.4, 129.0, 127.4, 117.4, 117.3, 107.0, 23.9; HRMS (ESI) calcd for C_13_H_11_N_2_^+^ [M+H^+^]: 195.0917; found: 195.0912.

*6-(4-Chlorophenyl)-2-methylnicotinonitrile* (**3e**). (31.9 mg, 70%). Isolated by flash column chromatography (eluent: ethyl acetate/petroleum ether = 1:20, R*_f_* = 0.3); white solid; ^1^H NMR (400 MHz, CDCl_3_) δ 7.99 (d, *J* = 8.7 Hz, 2H), 7.93 (d, *J* = 8.2 Hz, 1H), 7.63 (d, *J* = 8.2 Hz, 2H), 7.47 (d, *J* = 8.7 Hz, 2H), 2.83 (s, 3H); ^13^C NMR (100 MHz, CDCl_3_) *δ* 161.7, 158.4, 140.8, 136.7, 136.1, 129.2, 128.7, 117.2, 117.1, 107.3, 23.9; HRMS (ESI) calcd for C_13_H_10_ClN_2_Na^+^ [M+H^+^]: 229.0527; found: 229.0519.

*6-(4-Bromophenyl)-2-methylnicotinonitrile* (**3f**). (34.8 mg, 64%). Isolated by flash column chromatography (eluent: ethyl acetate/petroleum ether = 1:20, R*_f_* = 0.3); white solid; ^1^H NMR (400 MHz, CDCl_3_) *δ* 7.94–7.91 (m, 3H), 7.64–7.62 (m, 3H), 2.83 (s, 1H); ^13^C NMR (100 MHz, CDCl_3_) *δ* 161.7, 158.5, 140.8, 136.5, 132.2, 128.9, 125.1, 117.2, 117.0, 107.3, 23.9; HRMS (ESI) calcd for C_13_H_10_BrN_2_^+^ [M+H^+^]: 273.0022; found: 273.0032.

*6-(4-Fluorophenyl)-2-methylnicotinonitrile* (**3g**). (26.3 mg, 62%). Isolated by flash column chromatography (eluent: ethyl acetate/petroleum ether = 1:20, R*_f_* = 0.3); white solid; ^1^H NMR (400 MHz, CDCl_3_) *δ* 8.07–8.04 (m, 2H), 7.93 (d, *J* = 8.2 Hz, 1H), 7.61 (d, *J* = 8.2 Hz, 1H), 7.18 (t, *J* = 8.6 Hz, 2H), 2.83 (s, 3H); ^13^C NMR (100 MHz, CDCl_3_) *δ* 164.3 (d, *J* = 251.7 Hz), 161.7, 158.6, 140.8, 133.8, 129.4 (d, *J* = 8.6 Hz), 117.3, 117.0, 116.0 (d, *J* = 21.9 Hz), 23.9; ^19^F NMR (564 MHz, CDCl_3_) *δ* −110.4 (s, 1F); HRMS (ESI) calcd for C_13_H_10_FN_2_^+^ [M+H^+^]: 213.0832; found: 213.0818.

*6-(2-Chlorophenyl)-2-methylnicotinonitrile* (**3h**). (33.2 mg, 73%). Isolated by flash column chromatography (eluent: ethyl acetate/petroleum ether = 1:20, R*_f_* = 0.3); white solid; ^1^H NMR (400 MHz, CDCl_3_) *δ* 7.95 (d, *J* = 8.1 Hz, 1H), 7.62 (d, *J* = 8.6 Hz, 1H), 7.61–7.59 (m, 1H), 7.50–7.48 (m, 1H), 7.42–7.37 (m, 2H), 2.85 (s, 3H); ^13^C NMR (100 MHz, CDCl_3_) *δ* 161.5, 159.5, 139.8, 137.8, 132.1, 131.5, 130.6, 130.4, 127.3, 122.1, 117.1, 107.7, 23.8; HRMS (ESI) calcd for C_13_H_10_ClN_2_^+^ [M+H^+^]: 229.0527; found: 229.0520.

*6-(2,5-Dimethylphenyl)-2-methylnicotinonitrile* (**3i**). (29.3 mg, 66%). Isolated by flash column chromatography (eluent: ethyl acetate/petroleum ether = 1:20, R*_f_* = 0.4); white solid; ^1^H NMR (400 MHz, CDCl_3_) *δ* 7.90 (d, *J* = 8.0 Hz, 1H), 7.32 (d, *J* = 8.0 Hz, 1H), 7.19–7.12 (m, 3H), 2.82 (s, 3H), 2.34 (s, 3H), 2.30 (s, 3H); ^13^C NMR (100 MHz, CDCl_3_) *δ* 163.1, 161.2, 140.0, 138.7, 135.7, 132.7, 131.1, 130.1, 130.0, 121.2, 117.3, 106.7, 23.9, 20.9, 19.7; HRMS (ESI) calcd for C_15_H_15_N_2_^+^ [M+H^+^]: 223.1230; found: 223.1224.

*2-Methyl-6-(m-tolyl)nicotinonitrile* (**3j**). (26.3 mg, 63%). Isolated by flash column chromatography (eluent: ethyl acetate/petroleum ether = 1:20, R*_f_* = 0.4); white solid; ^1^H NMR (400 MHz, CDCl_3_) *δ* 7.91 (d, *J* = 8.2 Hz, 1H), 7.87 (s, 1H), 780 (d, *J* = 7.8 Hz, 1H), 7.64 (d, *J* = 8.2 Hz, 1H), 7.39 (t, *J* = 7.6 Hz, 1H), 7.29 (d, *J* = 7.5 Hz, 1H), 2.84 (s, 3H), 2.45 (s, 3H); ^13^C NMR (100 MHz, CDCl_3_) *δ* 161.5, 160.0, 140.6, 138.7, 137.7, 131.2, 128.9, 128.1, 124.5, 117.4, 106.8, 23.9, 21.5; HRMS (ESI) calcd for C_14_H_13_N_2_^+^ [M+H^+^]: 209.1073; found: 209.1068.

*6-(3-Chlorophenyl)-2-methylnicotinonitrile* (**3k**). (33.7 mg, 74%). Isolated by flash column chromatography (eluent: ethyl acetate/petroleum ether = 1:20, R*_f_* = 0.4); white solid; ^1^H NMR (400 MHz, CDCl_3_) *δ* 8.07–8.06 (m, 1H), 7.95 (d, *J* = 8.2 Hz, 1H), 7.92–7.89 (m, 1H), 7.64 (d, *J* = 8.2 Hz, 1H), 7.46–7.41 (m, 2H), 2.84 (s, 3H); ^13^C NMR (100 MHz, CDCl_3_) *δ* 161.8, 158.2, 140.9, 139.4, 135.2, 130.3, 130.2, 127.6, 125.4, 117.4, 117.1, 107.6, 23.9; HRMS (ESI) calcd for C_13_H_10_ClN_2_^+^ [M+H^+^]: 229.0527; found: 229.0519.

*2-Methyl-6-(naphthalen-2-yl)nicotinonitrile* (**3l**). (34.6 mg, 71%). Isolated by flash column chromatography (eluent: ethyl acetate/petroleum ether = 1:20, R*_f_* = 0.5); white solid; ^1^H NMR (400 MHz, CDCl_3_) *δ* 8.54 (s, 1H), 8.15 (dd, *J* = 1.8 Hz, 8.6 Hz, 1H), 7.95 (d, *J* = 8.1 Hz, 3H), 7.89–7.87 (m, 1H), 7.79 (d, *J* = 8.2 Hz, 1H), 7.57–7.53 (m, 2H), 2.88 (s, 3H); ^13^C NMR (100 MHz, CDCl_3_) *δ* 161.6, 159.6, 140.7, 134.9, 134.3, 133.3, 129.0, 128.8, 127.8, 127.6, 127.4, 126.7, 124.3, 117.5, 117.4, 106.9, 24.0; HRMS (ESI) calcd for C_17_H_13_N_2_^+^ [M + H^+^]: 245.1073; found: 245.1063.

*2,5-Dimethyl-6-phenylnicotinonitrile* (**3m**). (26.2 mg, 63%). Isolated by flash column chromatography (eluent: ethyl acetate/petroleum ether = 1:20, R*_f_* = 0.5); white solid; ^1^H NMR (400 MHz, CDCl_3_) *δ* 7.77 (s, 1H), 7.50–7.41 (m, 4H), 2.76 (s, 3H), 2.34 (s, 3H); ^13^C NMR (100 MHz, CDCl_3_) *δ* 161.6, 158.5, 142.1, 139.2, 128.9, 128.8, 128.5, 128.4, 117.3, 107.2, 23.3, 19.5; HRMS (ESI) calcd for C_14_H_13_N_2_^+^ [M+H^+^]: 209.1073; found: 209.1065.

*2-Phenyl-6-(p-tolyl)nicotinonitrile* (**3n**). (38.8 mg, 72%). Isolated by flash column chromatography (eluent: ethyl acetate/petroleum ether = 1:20, R*_f_* = 0.5); white solid; ^1^H NMR (400 MHz, CDCl_3_) *δ* 8.08–8.03 (m, 5H), 7.76 (d, *J* = 8.2 Hz, 1H), 7.57–7.50 (m, 3H), 7.31 (d, *J* = 8.0 Hz, 2H), 2.43 (s, 3H); ^13^C NMR (100 MHz, CDCl_3_) *δ* 160.5, 159.7, 142.4, 141.0, 137.6, 134.7, 130.2, 129.7, 129.0, 128.6, 127.4, 118.4, 117.4, 104.7, 21.5; HRMS (ESI) calcd for C_19_H_15_N_2_^+^ [M+H^+^]: 271.1230; found: 271.1219.

*1-(2-Methyl-6-(p-tolyl)pyridin-3-yl)ethan-1-one* (**3o**). (28.8 mg, 64%). Isolated by flash column chromatography (eluent: ethyl acetate/petroleum ether = 1:30, R*_f_* = 0.4); white solid; ^1^H NMR (400 MHz, CDCl_3_) *δ* 8.03 (d, *J* = 8.2 Hz, 1H), 7.96 (d, *J* = 8.1 Hz, 2H), 7.61 (d, *J* = 8.2 Hz, 1H), 7.28 (d, *J* = 8.1 Hz, 2H), 2.84 (s, 3H), 2.61 (s, 3H), 2.41 (s, 3H); ^13^C NMR (100 MHz, CDCl_3_) *δ* 200.0, 158.7, 158.6, 139.9, 138.0, 135.6, 130.4, 129.6, 127.2, 116.9, 29.3, 25.4, 21.4; HRMS (ESI) calcd for C_15_H_16_N_2_O^+^ [M+H^+^]: 226.1226; found: 226.1218.

*Methyl 2-methyl-6-(p-tolyl)nicotinate* (**3p**). (32.3 mg, 67%). Isolated by flash column chromatography (eluent: ethyl acetate/petroleum ether = 1:30, R*_f_* = 0.4); white solid; ^1^H NMR (400 MHz, CDCl_3_) *δ* 8.24 (d, *J* = 8.3 Hz, 1H), 7.96 (d, *J* = 8.2 Hz, 2H), 7.60 (d, *J* = 8.3 Hz, 1H), 7.28 (d, *J* = 8.2 Hz, 2H), 3.93 (m, 3H), 2.91 (s, 3H), 2.41 (s, 3H); ^13^C NMR (100 MHz, CDCl_3_) *δ* 167.1, 160.0, 159.2, 139.9, 139.3, 135.7, 129.6, 127.2, 122.9, 117.0, 52.1, 25.3, 21.4; HRMS (ESI) calcd for C_15_H_16_NO_2_^+^ [M+H^+^]: 242.1176; found: 242.1165.

## 4. Conclusions

In summary, we have developed a simple and efficient method for the synthesis of pyridines under a metal-free [3+3] annulation strategy of β,β-dichloromethyl peroxides and β-enaminonitriles. With this protocol, a series of polysubstituted pyridines were afforded in moderate to good yields under ambient conditions. The operational simplicity and good functional group compatibility would render this protocol a useful tool in organic synthesis.

## Figures and Tables

**Table 1:** Optimization studies ^a^.

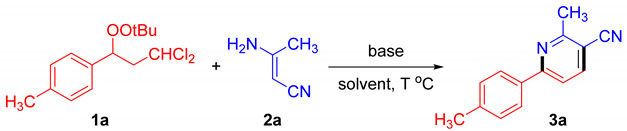					
Entry	Base	Solvent	T (^°^C)	1a Recovery (%) ^b^	3a Yield (%) ^b^
1	KOH	MeCN	25	31	55
2	NEt_3_	MeCN	25	87	N.D.
3	DIPEA	MeCN	25	89	N.D.
4	K_3_PO_4_	MeCN	25	94	N.D.
5	Cs_2_CO_3_	MeCN	25	91	trace
6	NaOH	MeCN	25	38	47
7	KOH	DMSO	25	trace	73
8	KOH	DMF	25	22	69
9	KOH	THF	25	50	37
10	KOH	MeOH	25	70	16
11	KOH	EA	25	90	N.D.
12	KOH	DCM	25	87	N.D.
13	KOH	DMSO	60	trace	71
14 ^c^	KOH	DMSO	25	37	52
15 ^d^	KOH	DMSO	25	trace	76

^a^ Reaction conditions: **1a** (0.2 mmol), **2a** (0.4 mmol), base (0.6 mmol), solvent (2.0 mL), 5 h, and under air. ^b^ Reported yields were based on **1a** and determined by ^1^H NMR using CH_2_Br_2_ as an internal standard. ^c^ **1a** (0.2 mmol), **2a** (0.4 mmol), base (0.4 mmol), solvent (2.0 mL), 5 h, and under air. ^d^ **1a** (0.2 mmol), **2a** (0.4 mmol), base (0.8 mmol), solvent (2.0 mL), 5 h, and under air.

**Table 2:** Scope of the substrates ^a^.

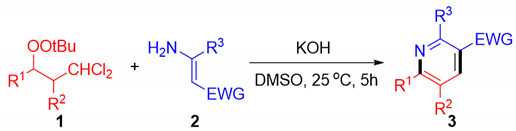
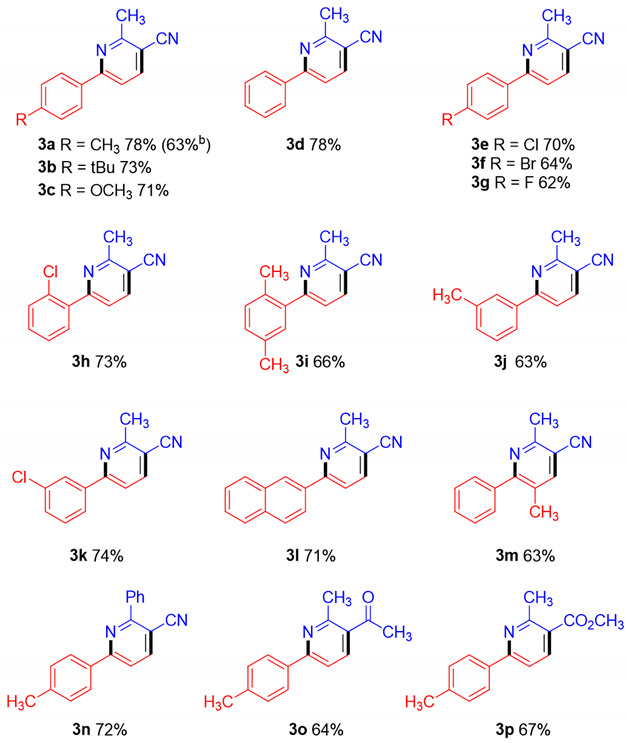

^a^ Reaction conditions: 1 (0.2 mmol), 2 (0.4 mmol), KOH (0.6 mmol), DMSO (2.0 mL), 25 °C, and 5 h unless otherwise noted. Reported yields were the isolated yields. ^b^ Reaction conditions: 1 (1.0 mmol), 2 (2.0 mmol), KOH (3.0 mmol), DMSO (10.0 mL), 25 °C, and 16 h.

## Data Availability

Data are contained within the article and Supplementary Materials (Table S1) and ^1^H and ^13^C spectra for **3**.

## Supplementary Materials

The following supporting information can be downloaded at: https://www.mdpi.com/article/10.3390/ijms26157105/s1.
