# Supplementary material for: Facile Synthesis of Polysubstituted Pyridines via Metal-Free [3+3] Annulation Between Enamines and β,β-Dichloromethyl Peroxides
Source: Int J Mol Sci. 2025 Jul 23;26(15):7105. doi: 10.3390/ijms26157105 (PMC12345729; doi:10.3390/ijms26157105)
Supplement: Supplementary file 1 [file ijms-26-07105-s001.zip › ijms-3740377-supplementary.pdf]

Supplementary Materials

Facile Synthesis of Polysubstituted Pyridines via Metal-free [3+3] Annulation between Enamines and β,β-Dichloromethyl Peroxides

Yangyang Ma [1], Hua Zhang [2], Zhonghao Zhou [3,*], Wenxiao Chang [1], Mohan Li [1], Yapei Zheng [1], Weizhuang Zhang [4], Huan Yue [1], Changdong Chen [1], Ming La [1,*] and Yongjun Han [1,*]

[1] College of Chemistry and Chemical Engineering, Pingdingshan University, Pingdingshan, Henan 467000, China

[2] College of Medicine, Pingdingshan University, Pingdingshan, Henan 467000, China

[3] School of Material Science and Engineering, Dalian Jiaotong University, Dalian 116028, China.

[4] Malaga School of Engineering, Pingdingshan University, Pingdingshan 467000, China

Correspondence: zhonghao_zhou@alu.ruc.edu.cn (Z. Z.); laming82@126.com (M. L.); 2773@pdsu.edu.cn (Y. H.)

Table of Contents

1. Starting materials S1

2. Copies of ^{1}H and ^{13}C spectra for **3** S2

1. Starting materials

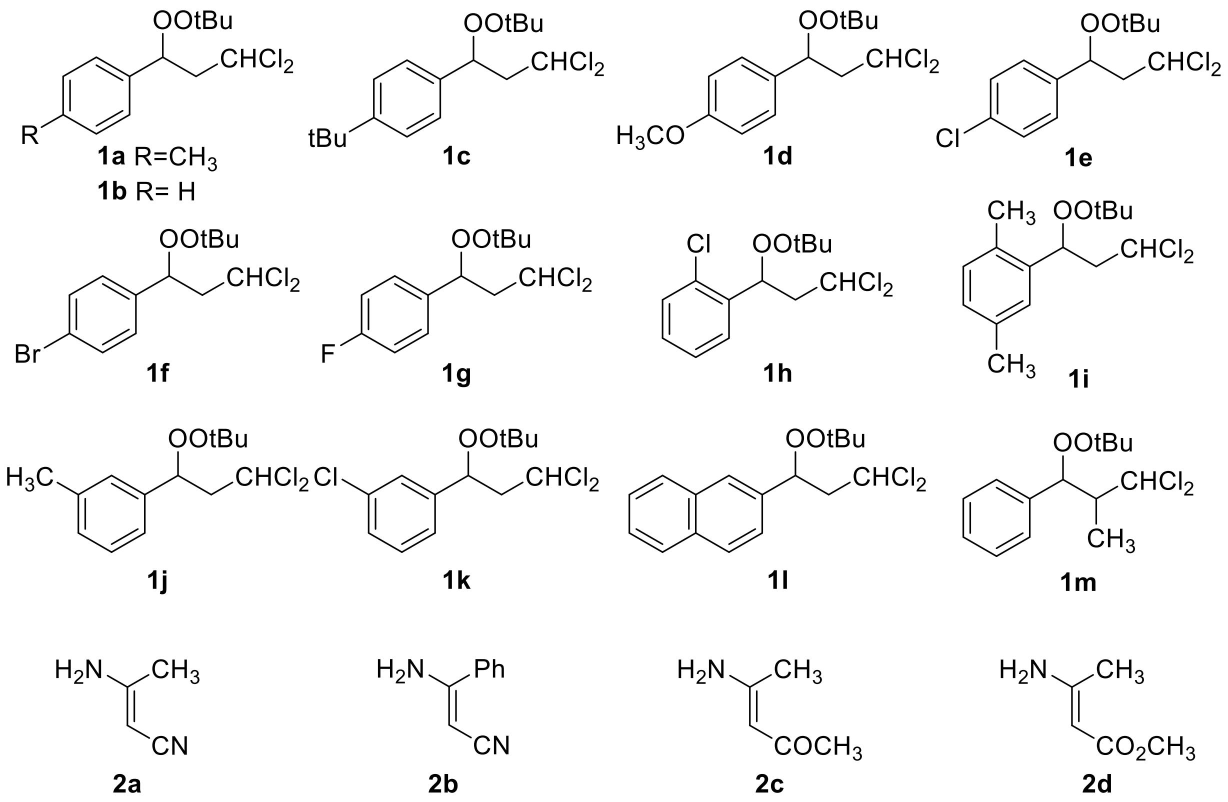

Figure S1. β,β-dichloro peroxides **1** and enamine **2**

2. Copies of ^{1}H and ^{13}C NMR spectra for 3

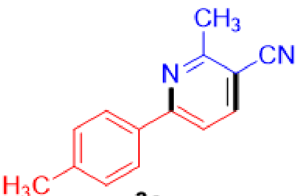

3a

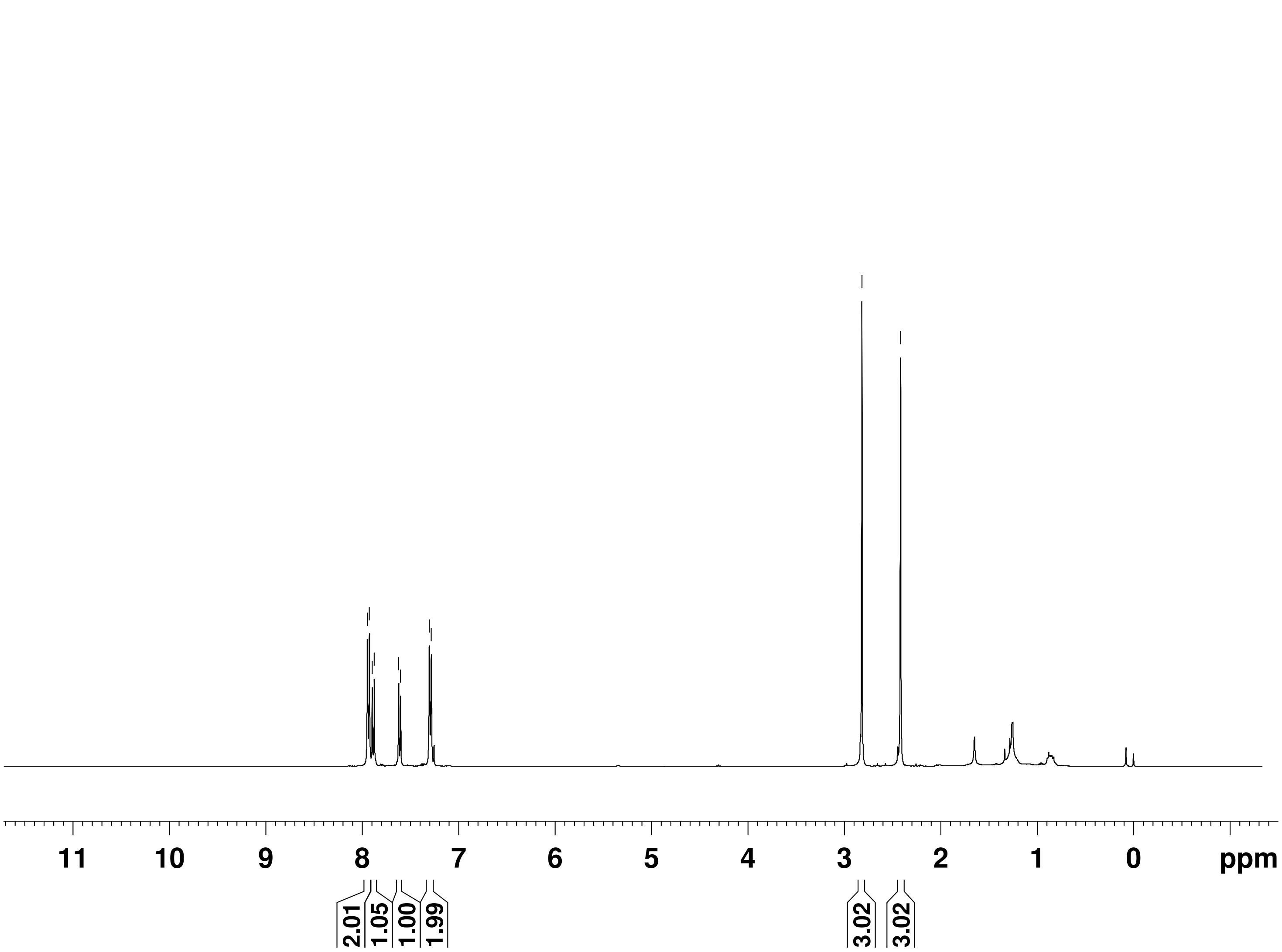

BRUKER

NAME	LV-MM-34-20240731
EXPNO	10
PROCNO	1
Date_	20240731
Time	20.55 h
INSTRUM	Avance
PROBHD	Z163739_0744 (
PULPROG	zg30
TD	65536
SOLVENT	CDCl3
NS	8
DS	0
SWH	6250.000 Hz
FIDRES	0.190735 Hz
AQ	5.2429299 sec
RG	71.8
DW	80.000 usec
DE	8.64 usec
TE	298.0 K
D1	1.00000000 sec
TD0	1
SFO1	400.1326008 MHz
NUC1	1H
P0	2.67 usec
P1	8.00 usec
SI	65536
SF	400.1300119 MHz
WDW	EM
SSB	0
LB	0.30 Hz
GB	0
PC	1.00

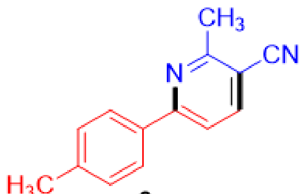

3a

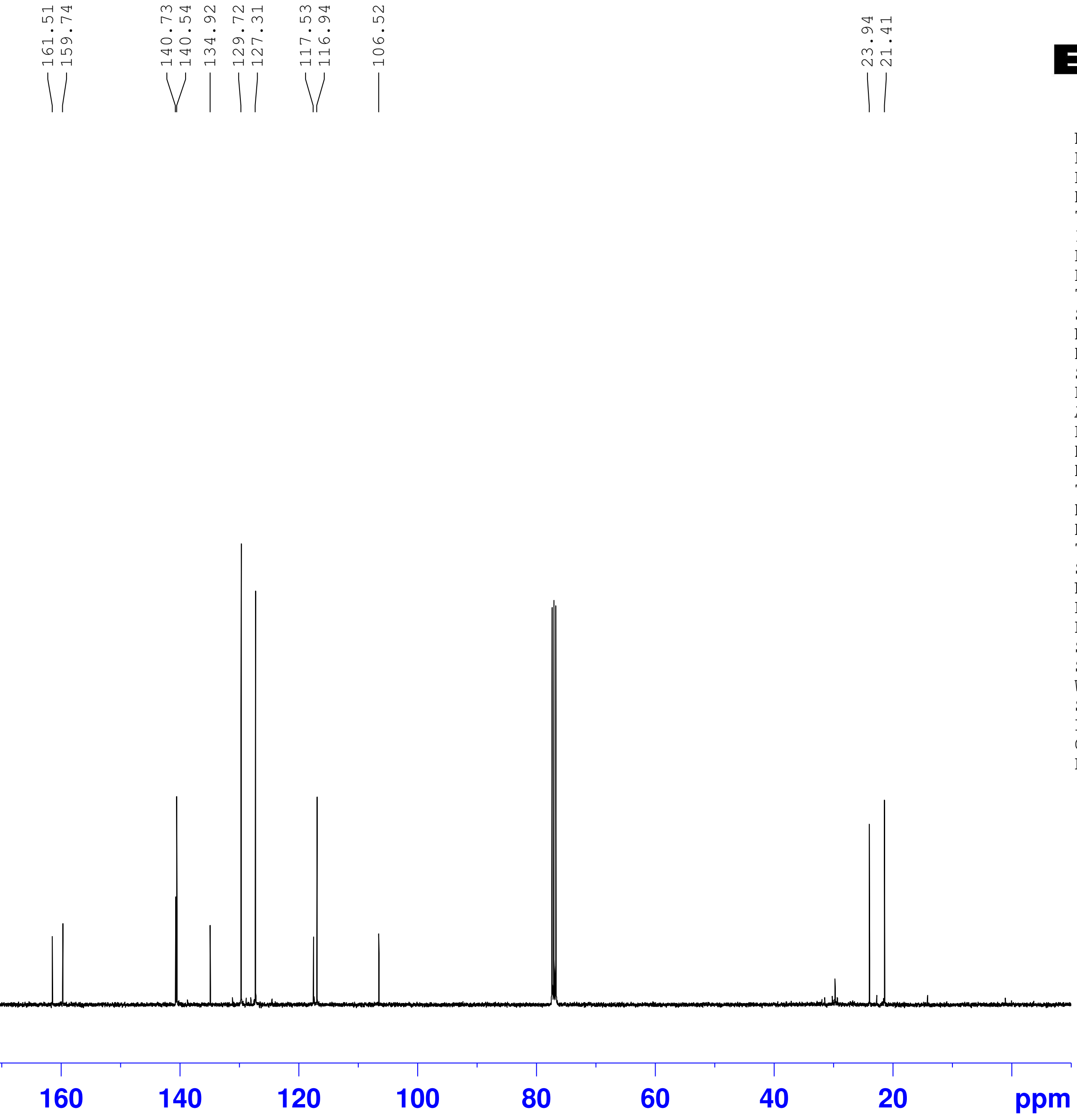

NAME	LV-MM-34-20240731
EXPNO	11
PROCNO	1
Date_	20240731
Time	21.42 h
INSTRUM	Avance
PROBHD	Z163739_0744 (
PULPROG	zgpg30
TD	65536
SOLVENT	CDCl3
NS	800
DS	4
SWH	23809.523 Hz
FIDRES	0.726609 Hz
AQ	1.3763061 se
RG	101
DW	21.000 us
DE	6.50 us
TE	298.0 K
D1	2.00000000 se
D11	0.03000000 se
TD0	1
SFO1	100.6228298 MH
NUC1	13C
P0	2.67 us
P1	8.00 us
SI	32768
SF	100.6127685 MH
WDW	EM
SSB	0
LB	1.00 Hz
GB	0
PC	1.40

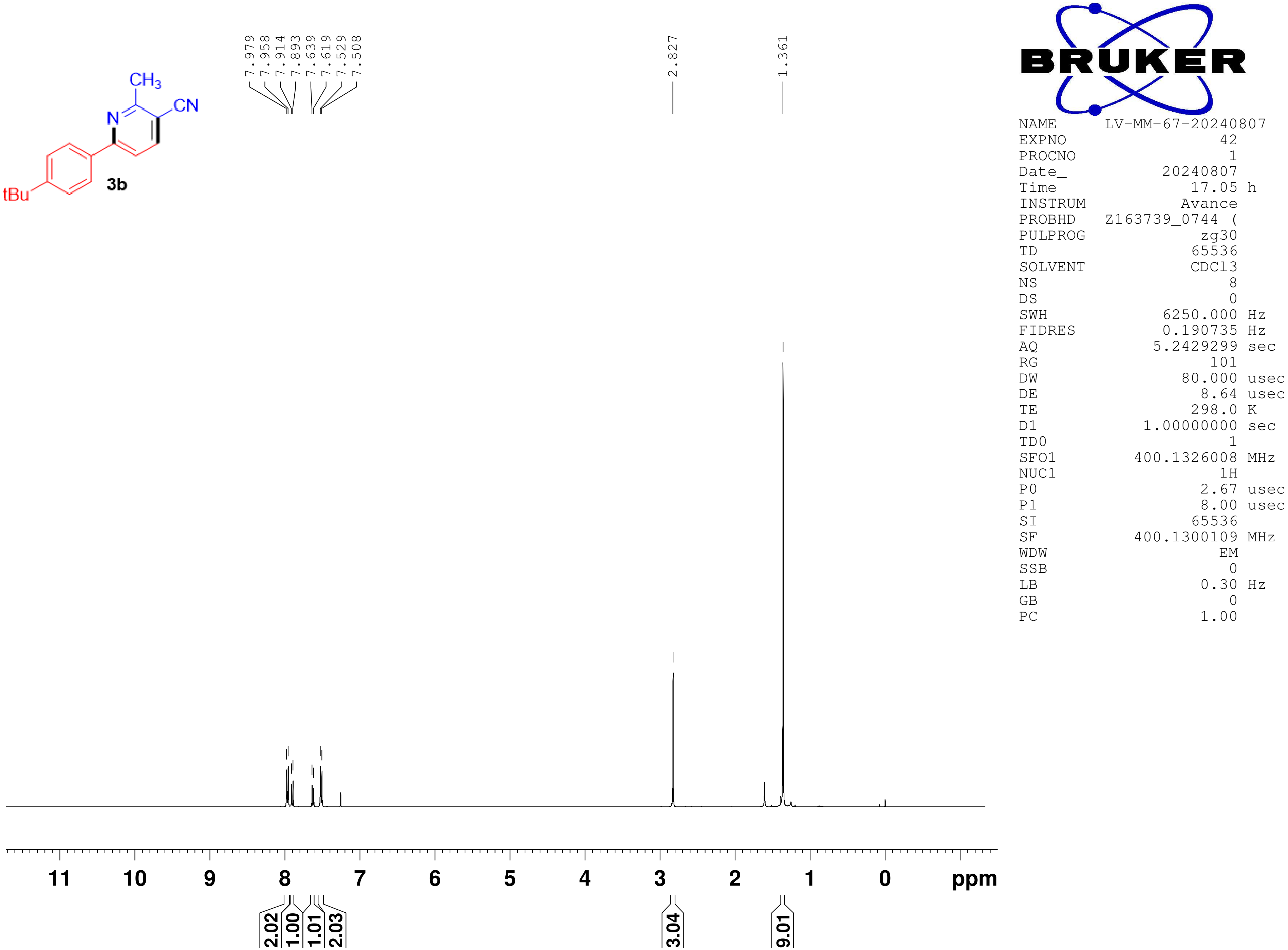
CH3
CN
N
tBu
3b
7.979
7.958
7.914
7.893
7.639
7.619
7.529
7.508
2.827
1.361
NAME LV-MM-67-20240807
EXPNO 42
PROCNO 1
Date_ 20240807
Time 17.05 h
INSTRUM Avance
PROBHD Z163739_0744 (
PULPROG zg30
TD 65536
SOLVENT CDCl3
NS 8
DS 0
SWH 6250.000 Hz
FIDRES 0.190735 Hz
AQ 5.2429299 sec
RG 101
DW 80.000 usec
DE 8.64 usec
TE 298.0 K
D1 1.00000000 sec
TD0 1
SFO1 400.1326008 MHz
NUC1 1H
P0 2.67 usec
P1 8.00 usec
SI 65536
SF 400.1300109 MHz
WDW EM
SSB 0
LB 0.30 Hz
GB 0
PC 1.00
11 10 9 8 7 6 5 4 3 2 1 0 ppm
2.02
1.00
1.01
2.03
3.04
9.01

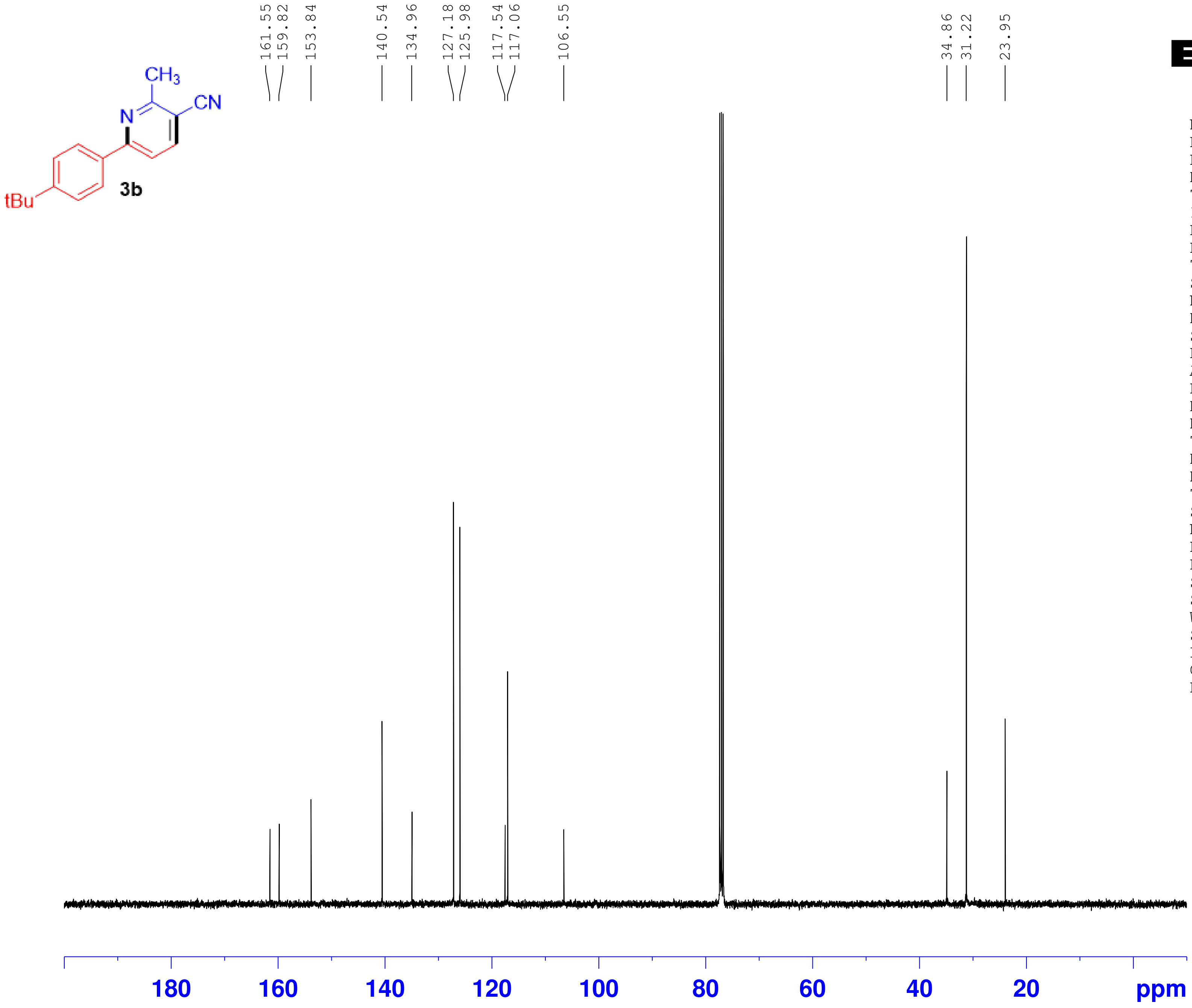

3b

NAME	LV-MM-67-20240807
EXPNO	43
PROCNO	1
Date_	20240807
Time	17.52 h
INSTRUM	Avance
PROBHD	Z163739_0744 (
PULPROG	zgpg30
TD	65536
SOLVENT	CDCl3
NS	800
DS	4
SWH	23809.523 Hz
FIDRES	0.726609 Hz
AQ	1.3763061 se
RG	101
DW	21.000 us
DE	6.50 us
TE	298.0 K
D1	2.00000000 se
D11	0.03000000 se
TD0	1
SFO1	100.6228298 MH
NUC1	13C
P0	2.67 us
P1	8.00 us
SI	32768
SF	100.6127685 MH
WDW	EM
SSB	0
LB	1.00 Hz
GB	0
PC	1.40

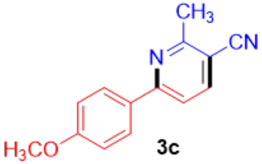

3c

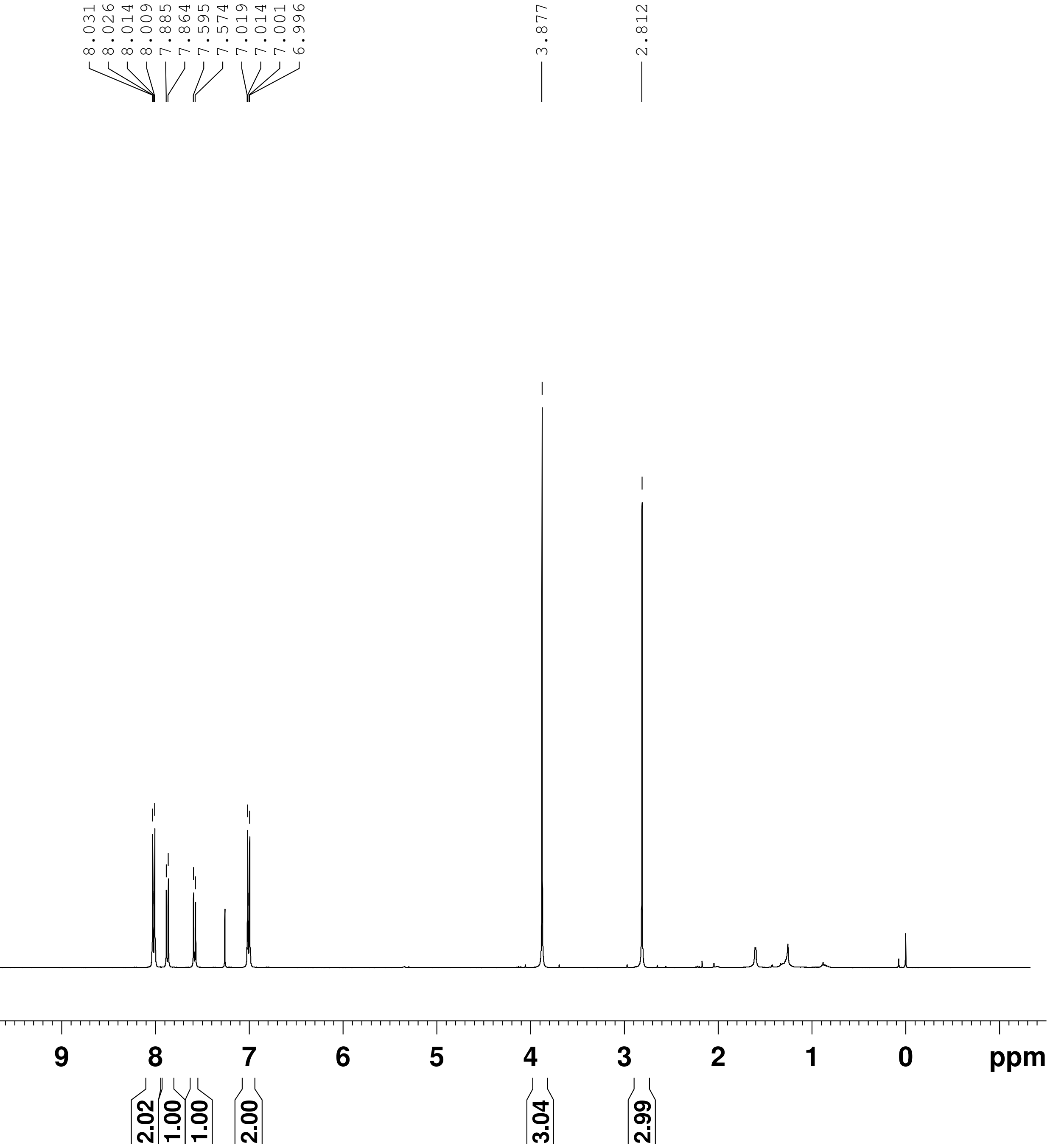

NAME	LV-MM-49-20240806
EXPNO	10
PROCNO	1
Date_	20240806
Time	1.53 h
INSTRUM	Avance
PROBHD	Z163739_0744 (
PULPROG	zg30
TD	65536
SOLVENT	CDCl3
NS	8
DS	0
SWH	6250.000 Hz
FIDRES	0.190735 Hz
AQ	5.2429299 sec
RG	101
DW	80.000 usec
DE	8.64 usec
TE	298.0 K
D1	1.00000000 sec
TD0	1
SFO1	400.1326008 MHz
NUC1	1H
P0	2.67 usec
P1	8.00 usec
SI	65536
SF	400.1300097 MHz
WDW	EM
SSB	0
LB	0.30 Hz
GB	0
PC	1.00

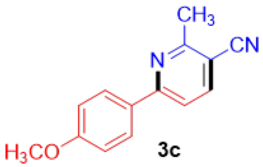

3c

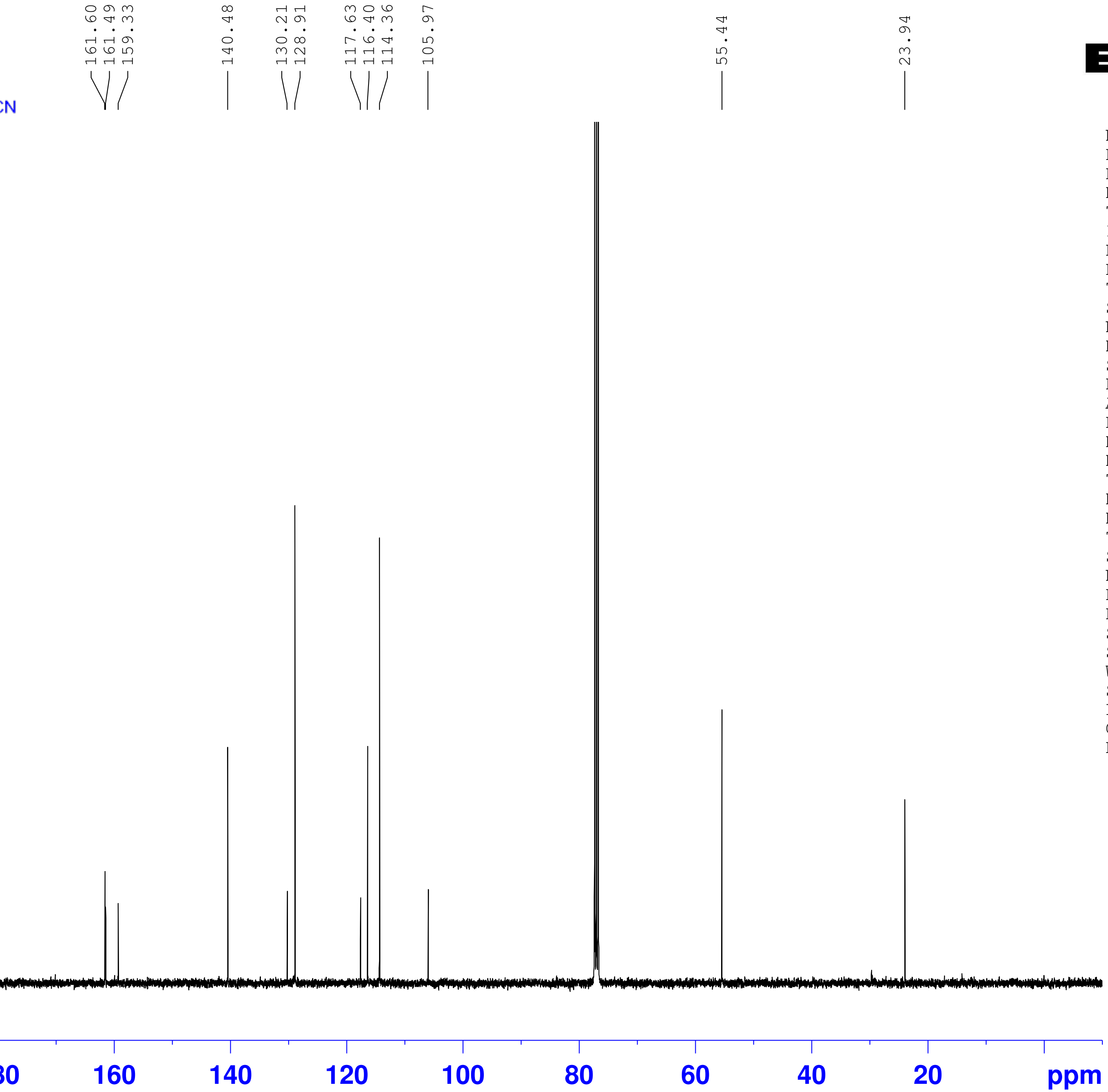

NAME	LV-MM-49-20240806
EXPNO	11
PROCNO	1
Date_	20240806
Time	2.52 h
INSTRUM	Avance
PROBHD	Z163739_0744 (
PULPROG	zgpg30
TD	65536
SOLVENT	CDCl3
NS	1024
DS	4
SWH	23809.523 Hz
FIDRES	0.726609 Hz
AQ	1.3763061 se
RG	101
DW	21.000 us
DE	6.50 us
TE	298.0 K
D1	2.00000000 se
D11	0.03000000 se
TD0	1
SFO1	100.6228298 MH
NUC1	13C
P0	2.67 us
P1	8.00 us
SI	32768
SF	100.6127685 MH
WDW	EM
SSB	0
LB	1.00 Hz
GB	0
PC	1.40

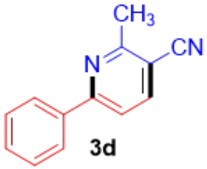

3d

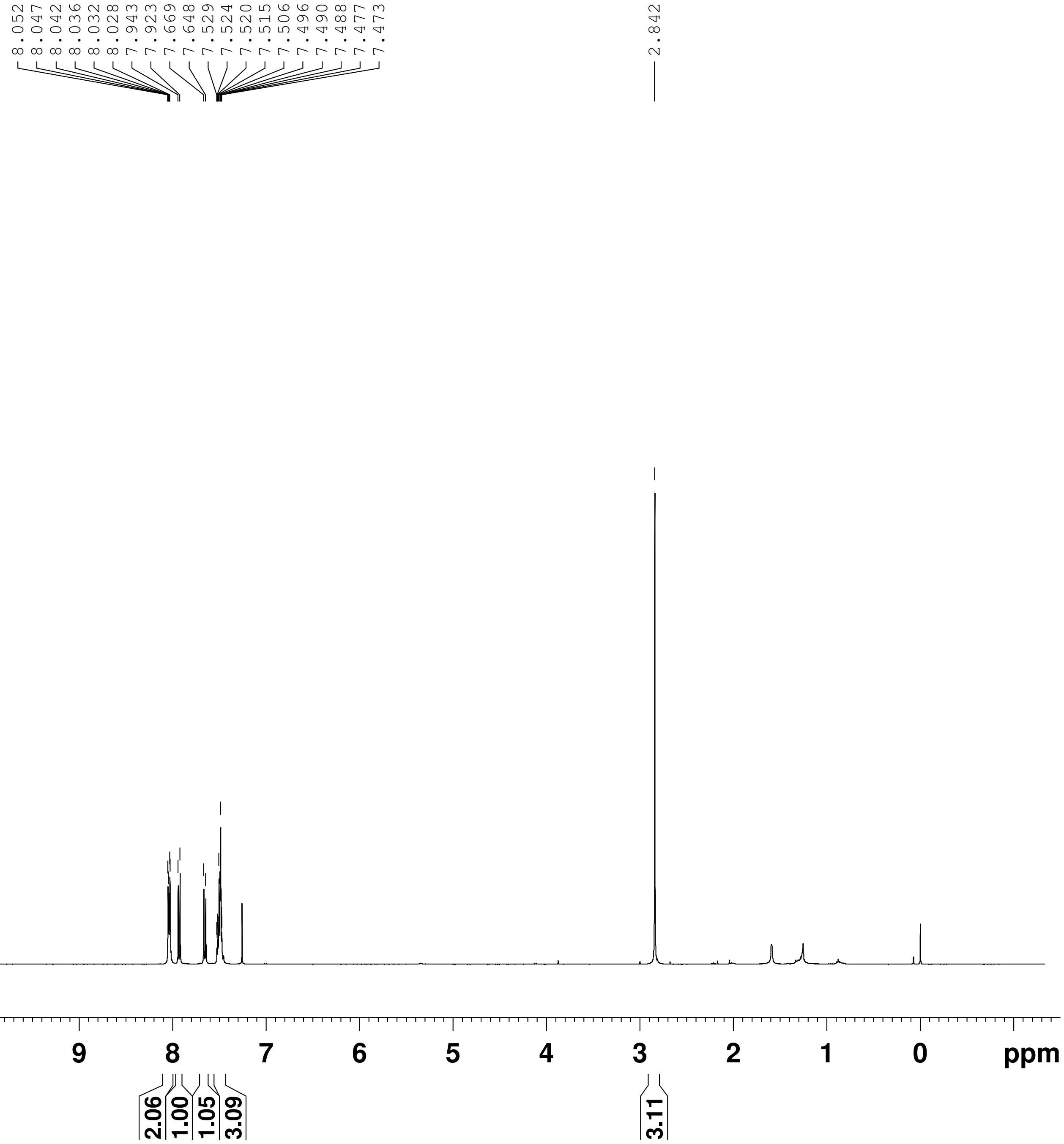

BRUKER

NAME	LV-MM-57-20240806
EXPNO	10
PROCNO	1
Date_	20240806
Time	0.19 h
INSTRUM	Avance
PROBHD	Z163739_0744 (
PULPROG	zg30
TD	65536
SOLVENT	CDCl3
NS	8
DS	0
SWH	6250.000 Hz
FIDRES	0.190735 Hz
AQ	5.2429299 sec
RG	101
DW	80.000 usec
DE	8.64 usec
TE	298.0 K
D1	1.00000000 sec
TD0	1
SFO1	400.1326008 MHz
NUC1	1H
P0	2.67 usec
P1	8.00 usec
SI	65536
SF	400.1300104 MHz
WDW	EM
SSB	0
LB	0.30 Hz
GB	0
PC	1.00

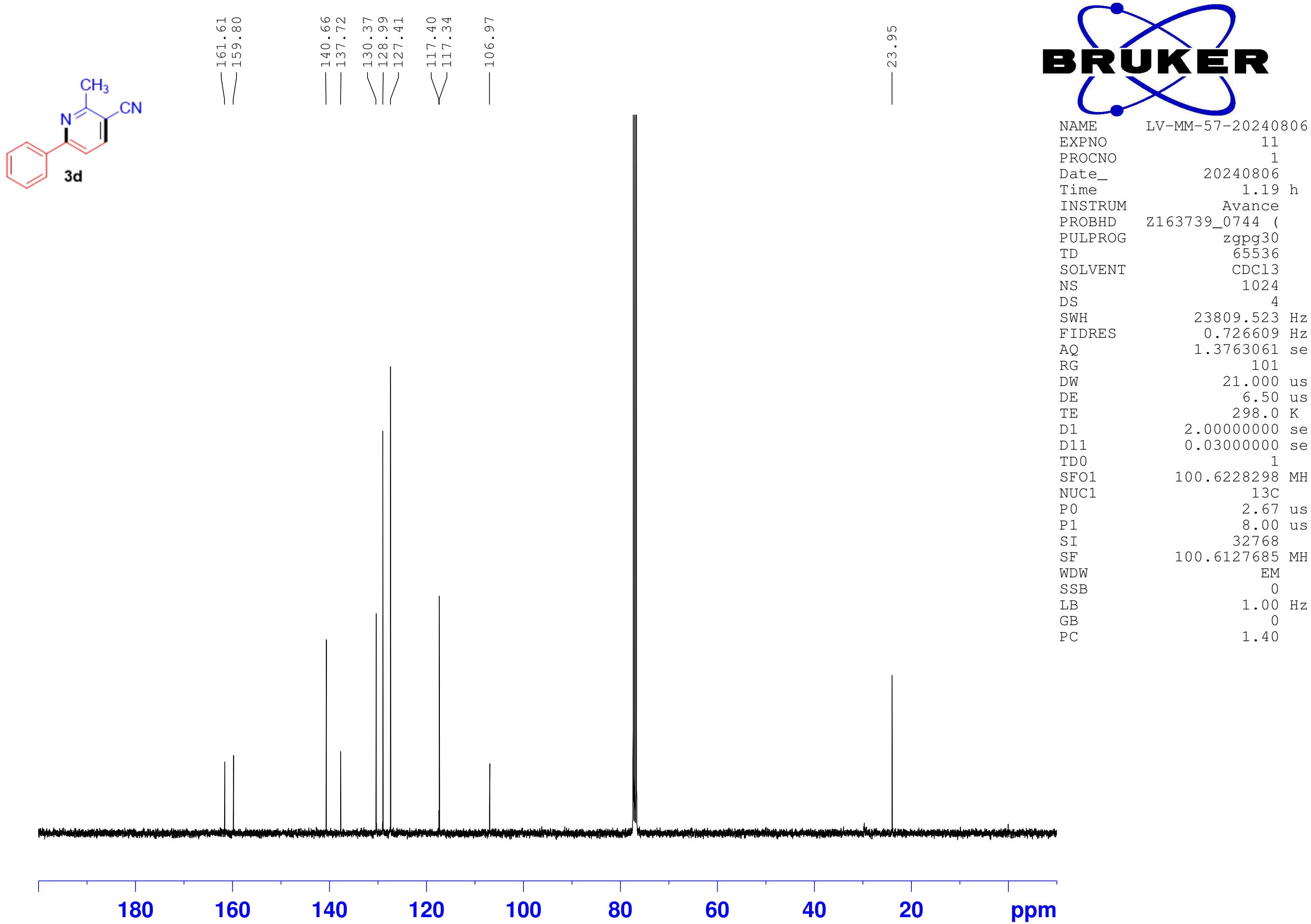

CH3
CN
N
3d
161.61
159.80
140.66
137.72
130.37
128.99
127.41
117.40
117.34
106.97
23.95
BRUKER
NAME LV-MM-57-20240806
EXPNO 11
PROCNO 1
Date_ 20240806
Time 1.19 h
INSTRUM Avance
PROBHD Z163739_0744 (
PULPROG zgpg30
TD 65536
SOLVENT CDCl3
NS 1024
DS 4
SWH 23809.523 Hz
FIDRES 0.726609 Hz
AQ 1.3763061 se
RG 101
DW 21.000 us
DE 6.50 us
TE 298.0 K
D1 2.00000000 se
D11 0.03000000 se
TD0 1
SFO1 100.6228298 MH
NUC1 13C
P0 2.67 us
P1 8.00 us
SI 32768
SF 100.6127685 MH
WDW EM
SSB 0
LB 1.00 Hz
GB 0
PC 1.40
180
160
140
120
100
80
60
40
20
ppm

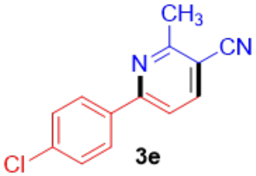

3e

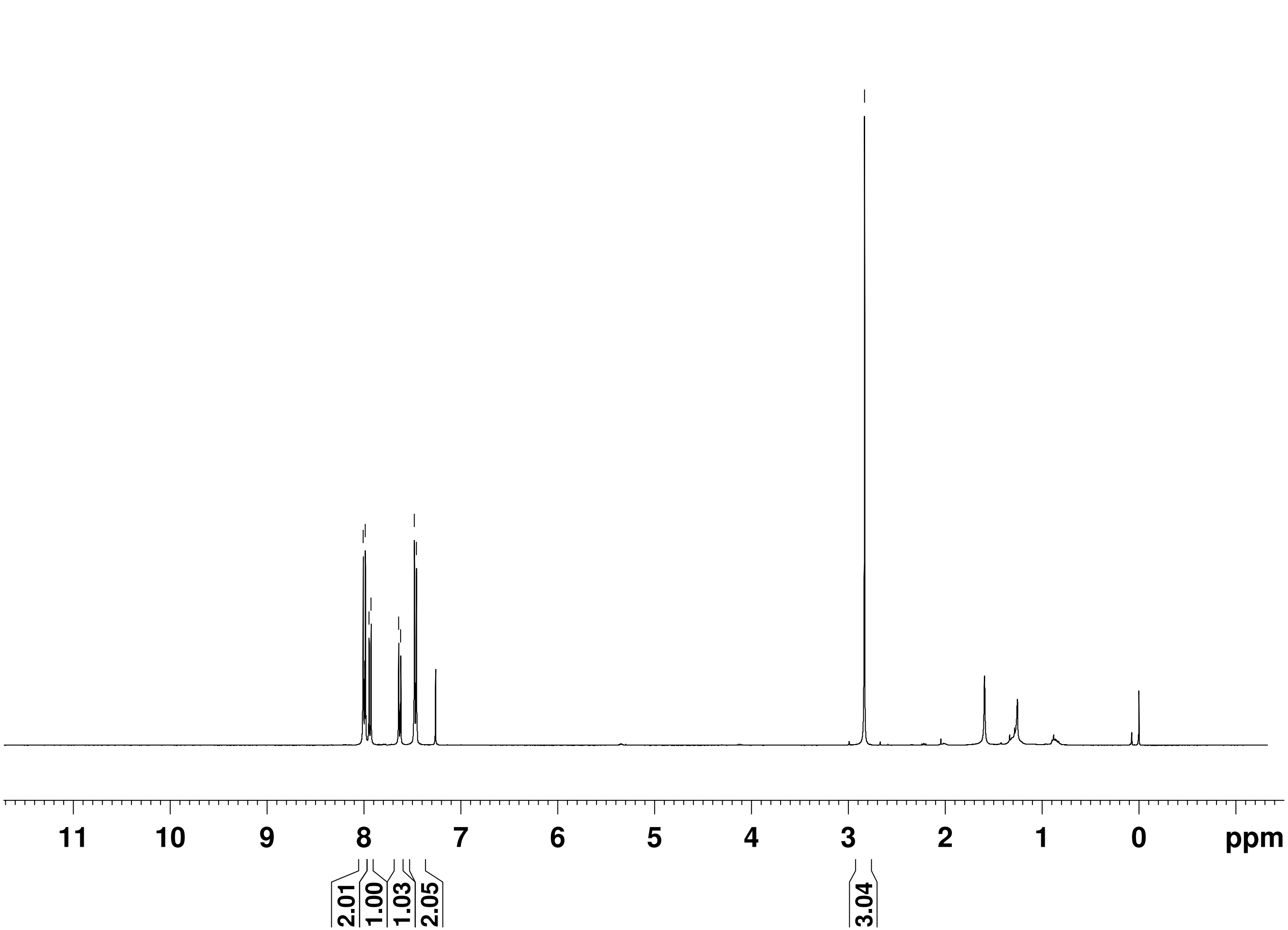

NAME	LV-MM-48-20240804
EXPNO	43
PROCNO	1
Date_	20240805
Time	1.51 h
INSTRUM	Avance
PROBHD	Z163739_0744 (
PULPROG	zg30
TD	65536
SOLVENT	CDCl3
NS	8
DS	0
SWH	6250.000 Hz
FIDRES	0.190735 Hz
AQ	5.2429299 sec
RG	101
DW	80.000 usec
DE	8.64 usec
TE	298.0 K
D1	1.00000000 sec
TD0	1
SFO1	400.1326008 MHz
NUC1	1H
P0	2.67 usec
P1	8.00 usec
SI	65536
SF	400.1300093 MHz
WDW	EM
SSB	0
LB	0.30 Hz
GB	0
PC	1.00

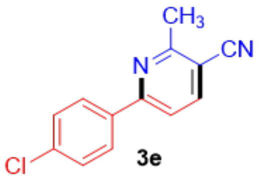

3e

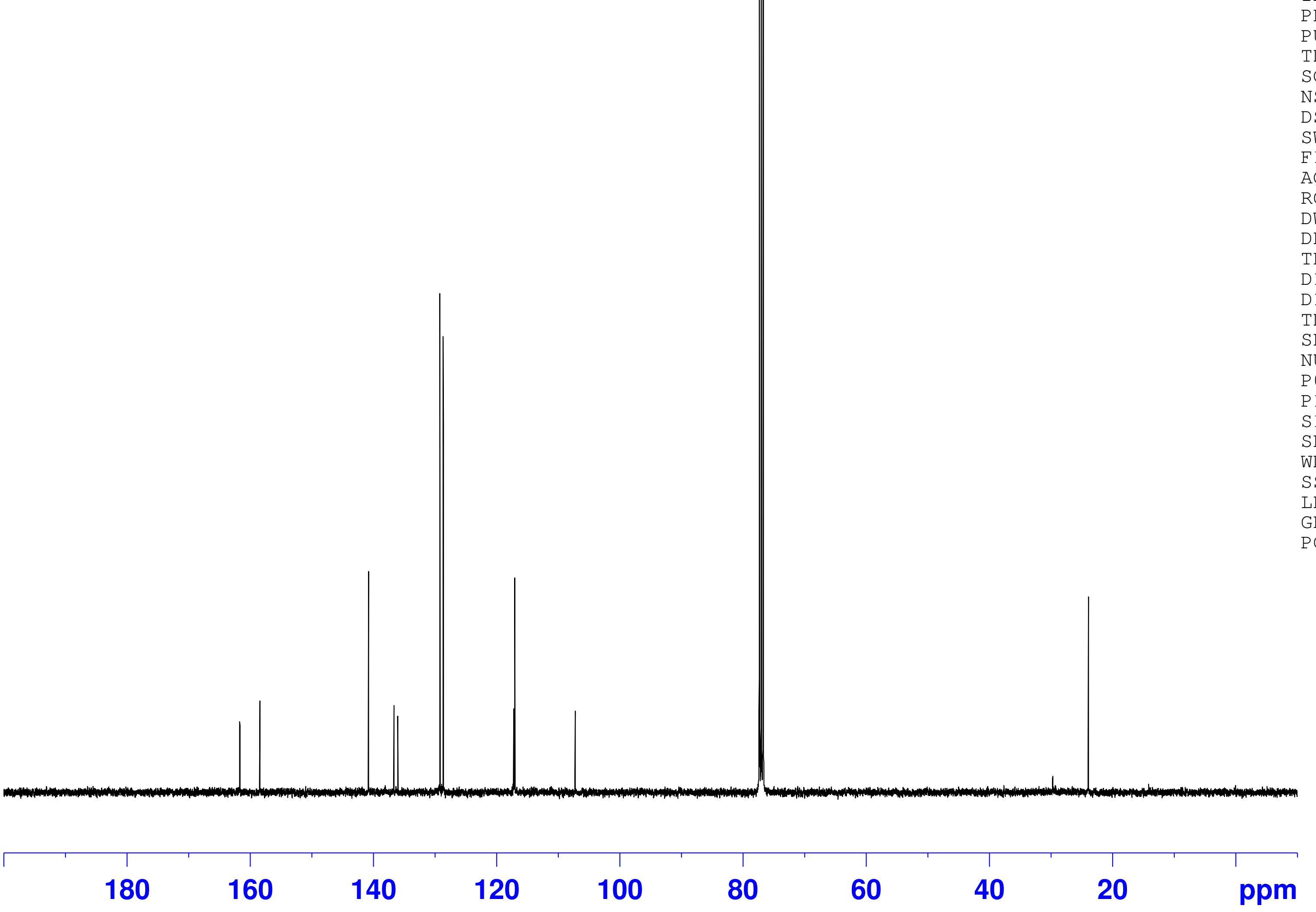

NAME	LV-MM-48-20240804
EXPNO	42
PROCNO	1
Date_	20240805
Time	1.49 h
INSTRUM	Avance
PROBHD	Z163739_0744 (
PULPROG	zgpg30
TD	65536
SOLVENT	CDCl3
NS	1024
DS	4
SWH	23809.523 Hz
FIDRES	0.726609 Hz
AQ	1.3763061 se
RG	101
DW	21.000 us
DE	6.50 us
TE	298.0 K
D1	2.00000000 se
D11	0.03000000 se
TD0	1
SFO1	100.6228298 MH
NUC1	13C
P0	2.67 us
P1	8.00 us
SI	32768
SF	100.6127685 MH
WDW	EM
SSB	0
LB	1.00 Hz
GB	0
PC	1.40

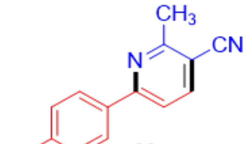

3f

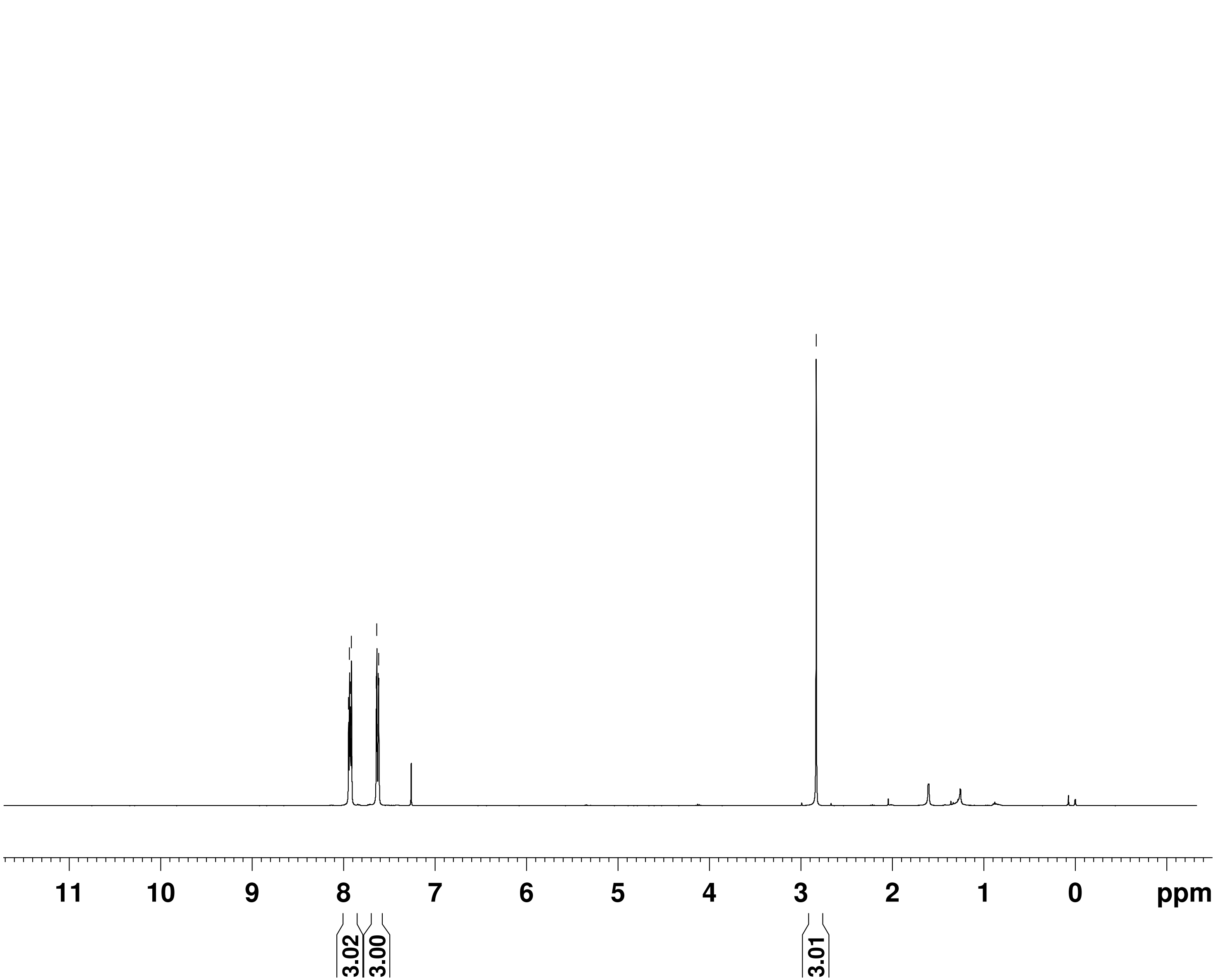

BRUKER

NAME	LV-MM-69-20240807	
EXPNO	52	
PROCNO	1	
Date_	20240807	
Time	17.57	h
INSTRUM	Avance	
PROBHD	Z163739_0744 (	
PULPROG	zg30	
TD	65536	
SOLVENT	CDCl3	
NS	8	
DS	0	
SWH	6250.000	Hz
FIDRES	0.190735	Hz
AQ	5.2429299	sec
RG	101	
DW	80.000	usec
DE	8.64	usec
TE	298.0	K
D1	1.00000000	sec
TD0	1	
SFO1	400.1326008	MHz
NUC1	1H	
P0	2.67	usec
P1	8.00	usec
SI	65536	
SF	400.1300092	MHz
WDW	EM	
SSB	0	
LB	0.30	Hz
GB	0	
PC	1.00	

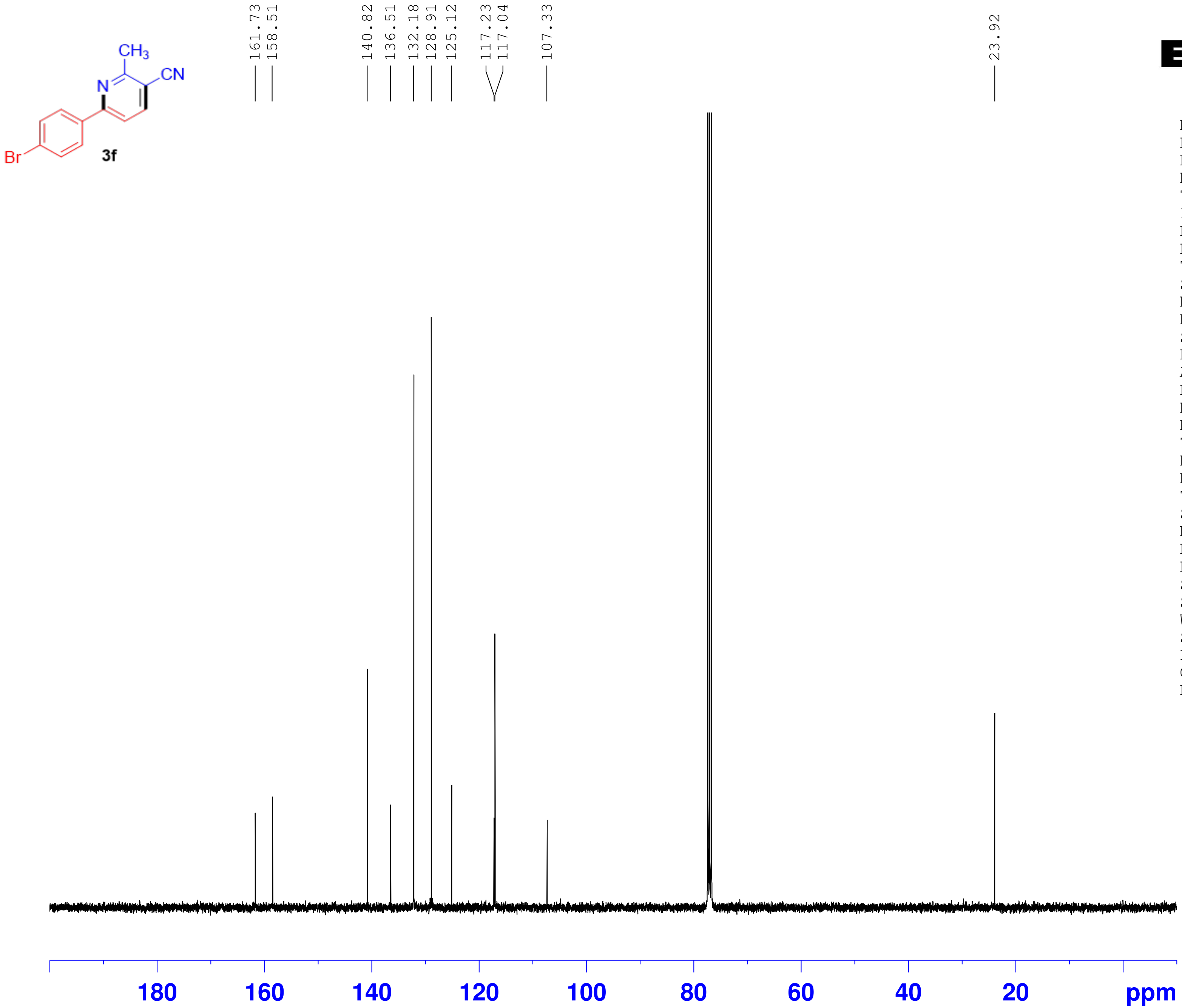

3f

NAME	LV-MM-69-20240807
EXPNO	53
PROCNO	1
Date_	20240807
Time	18.45 h
INSTRUM	Avance
PROBHD	Z163739_0744 (
PULPROG	zgpg30
TD	65536
SOLVENT	CDCl3
NS	800
DS	4
SWH	23809.523 Hz
FIDRES	0.726609 Hz
AQ	1.3763061 se
RG	101
DW	21.000 us
DE	6.50 us
TE	298.0 K
D1	2.00000000 se
D11	0.03000000 se
TD0	1
SFO1	100.6228298 MH
NUC1	13C
P0	2.67 us
P1	8.00 us
SI	32768
SF	100.6127685 MH
WDW	EM
SSB	0
LB	1.00 Hz
GB	0
PC	1.40

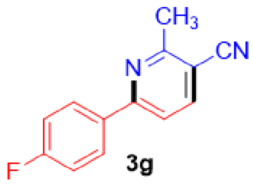

3g

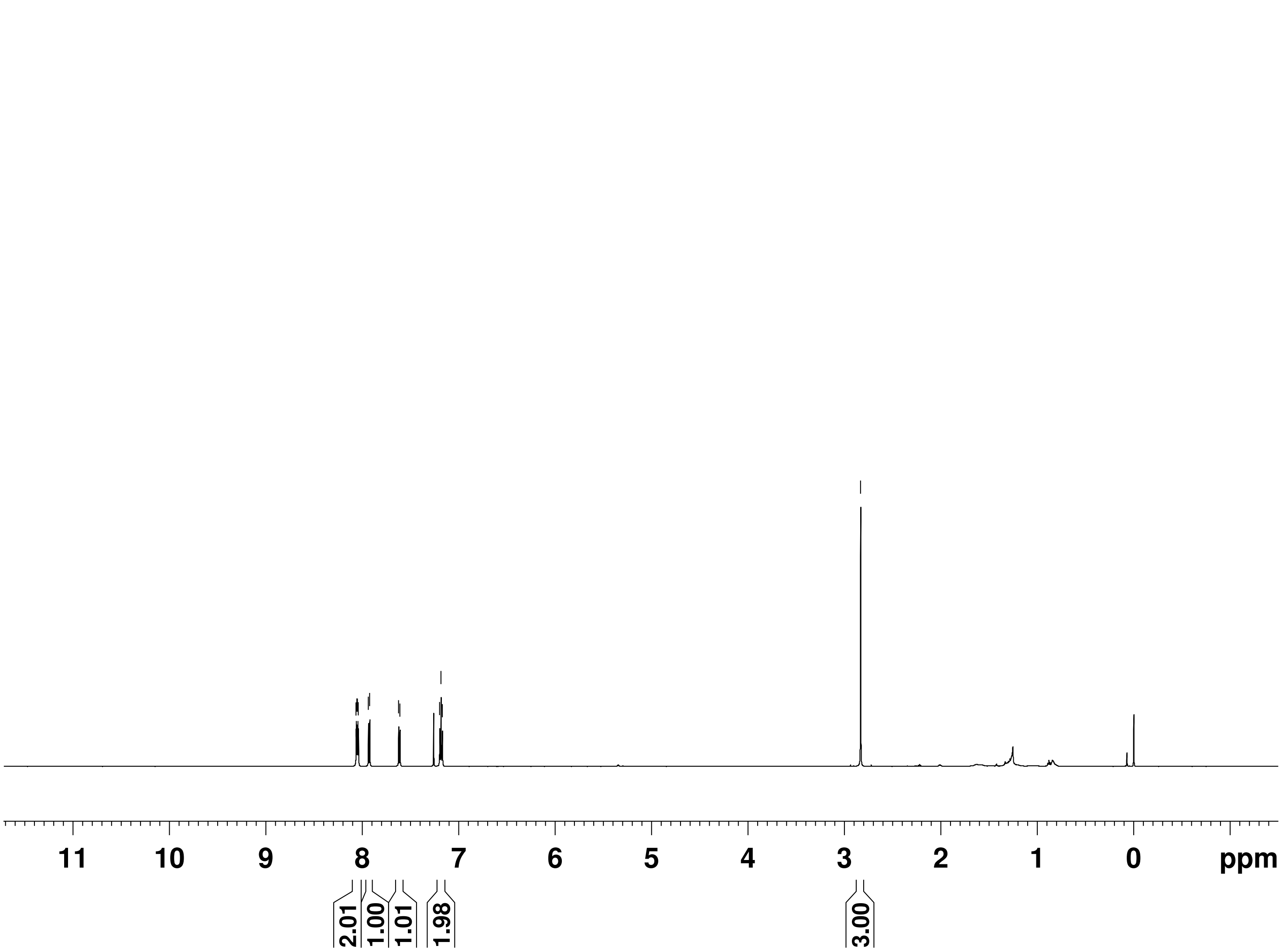

NAME	LV-LCH-1000P-20250415
EXPNO	1
PROCNO	1
Date_	20250415
Time	21.50
INSTRUM	spect
PROBHD	5 mm PABBO BB/
PULPROG	zg30
TD	65536
SOLVENT	CDCl3
NS	8
DS	0
SWH	9615.385 Hz
FIDRES	0.146719 Hz
AQ	3.4079220 sec
RG	124.15
DW	52.000 usec
DE	6.50 usec
TE	298.0 K
D1	1.00000000 sec
TD0	1

======== CHANNEL f1 ========

SFO1	600.1739011 MHz
NUC1	1H
P1	9.96 usec
SI	65536
SF	600.1700147 MHz
WDW	EM
SSB	0
LB	0.30 Hz
GB	0
PC	1.00

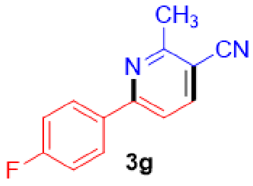

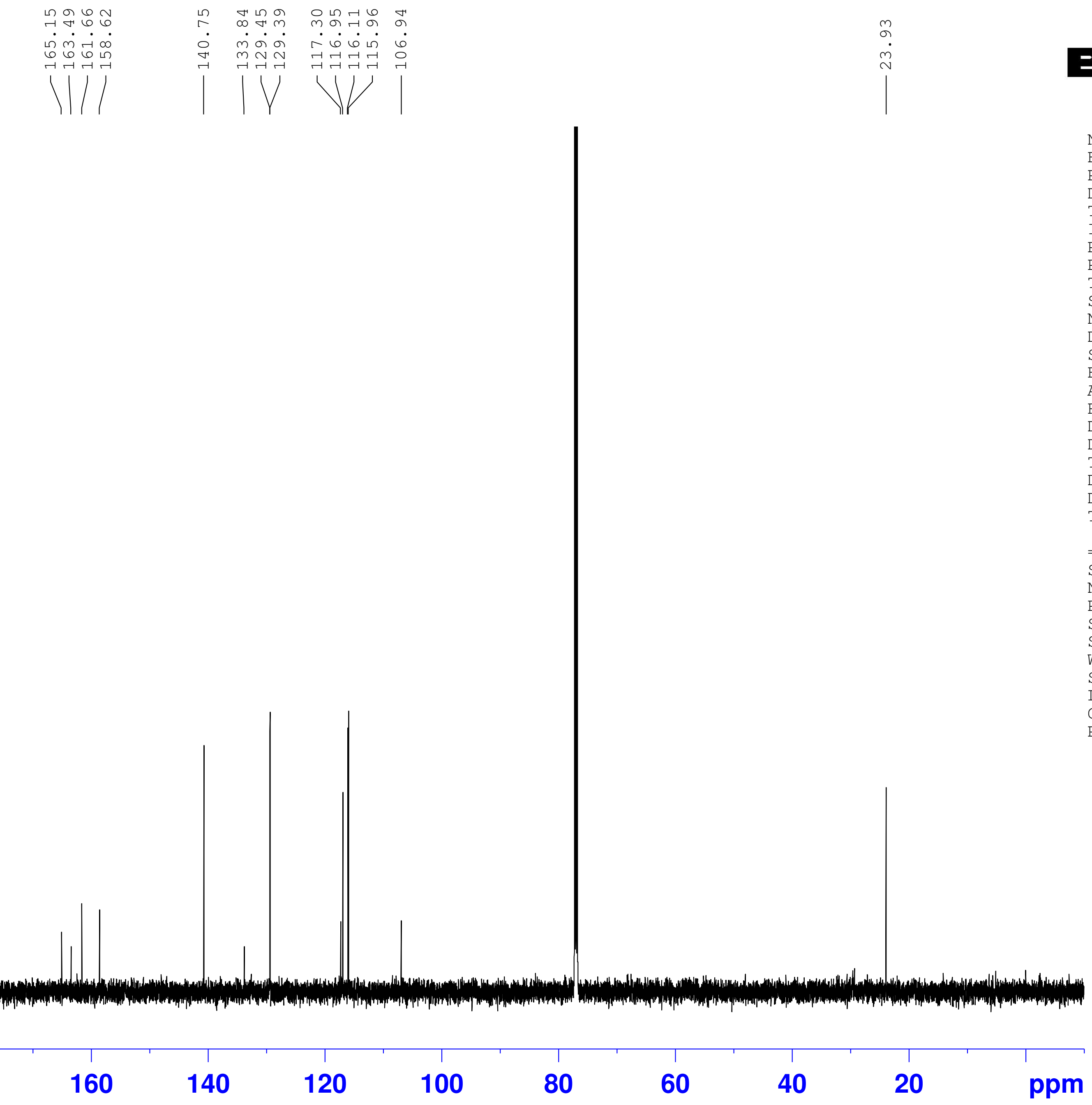

BRUKER

NAME	LV-LCH-1000P-202504
EXPNO	3
PROCNO	1
Date_	20250415
Time	22.13
INSTRUM	spect
PROBHD	5 mm PABBO BB/
PULPROG	zgpg30
TD	65536
SOLVENT	CDCl3
NS	400
DS	4
SWH	36057.691 Hz
FIDRES	0.550197 Hz
AQ	0.9088159 sec
RG	190.02
DW	13.867 usec
DE	6.50 usec
TE	298.0 K
D1	2.00000000 sec
D11	0.03000000 sec
TD0	1

======== CHANNEL f1 ========

SFO1	150.9279571 MHz
NUC1	13C
P1	11.90 usec
SI	32768
SF	150.9128665 MHz
WDW	EM
SSB	0
LB	1.00 Hz
GB	0
PC	1.40

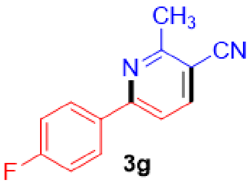

3g

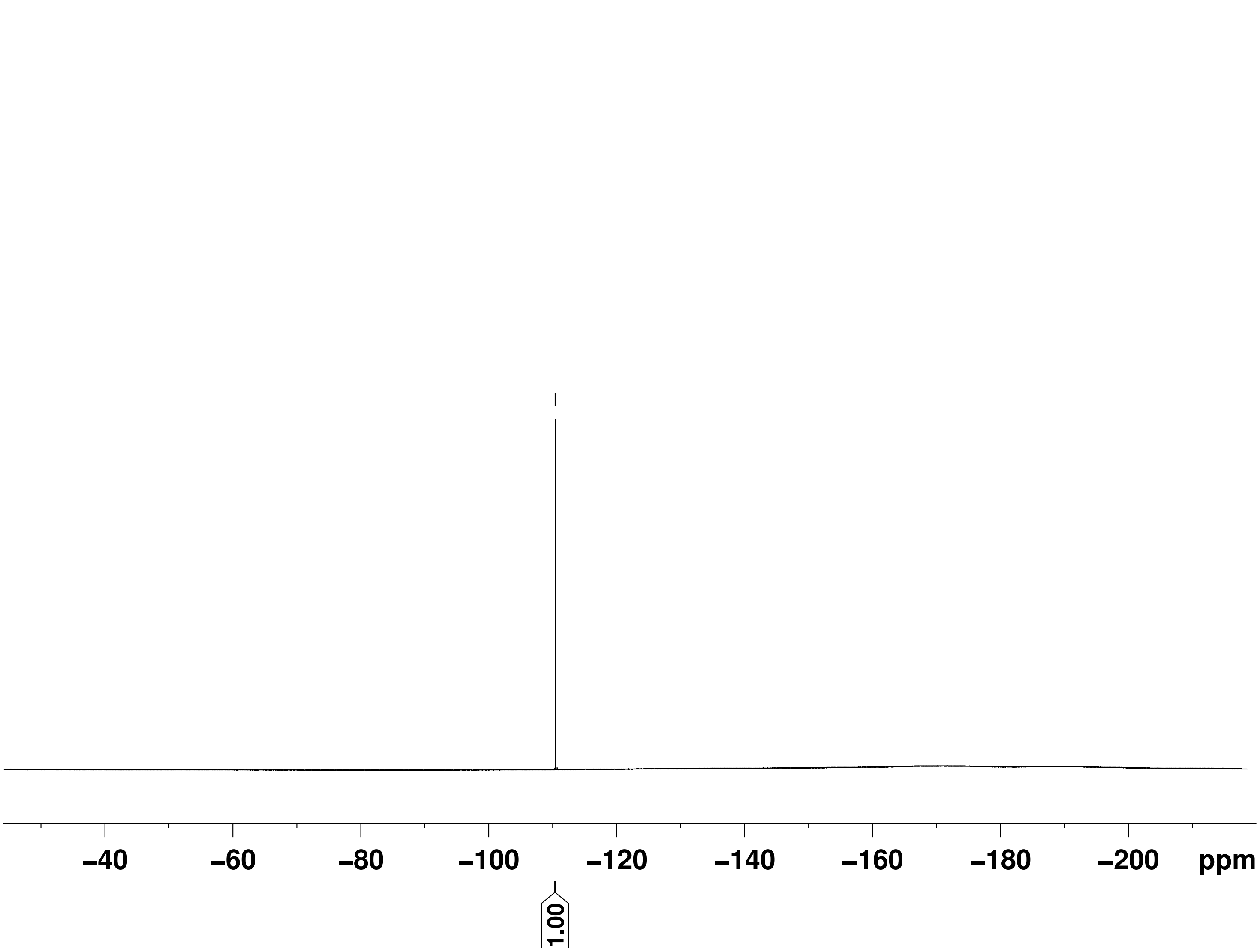

NAME	LV-LCH-1000P-20250415
EXPNO	2
PROCNO	1
Date_	20250415
Time	21.52
INSTRUM	spect
PROBHD	5 mm PABBO BB/
PULPROG	zgfhigqn.2
TD	131072
SOLVENT	CDCl3
NS	16
DS	4
SWH	133928.578 Hz
FIDRES	1.021794 Hz
AQ	0.4893855 sec
RG	190.02
DW	3.733 usec
DE	6.50 usec
TE	298.1 K
D1	1.00000000 sec
D11	0.03000000 sec
D12	0.00002000 sec
TD0	1

======== CHANNEL f1 ========

SFO1	564.6675534 MHz
NUC1	19F
P1	12.00 usec
SI	65536
SF	564.7240258 MHz
WDW	EM
SSB	0
LB	0.30 Hz
GB	0
PC	1.00

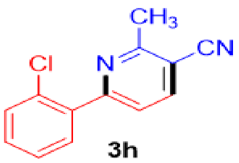

3h

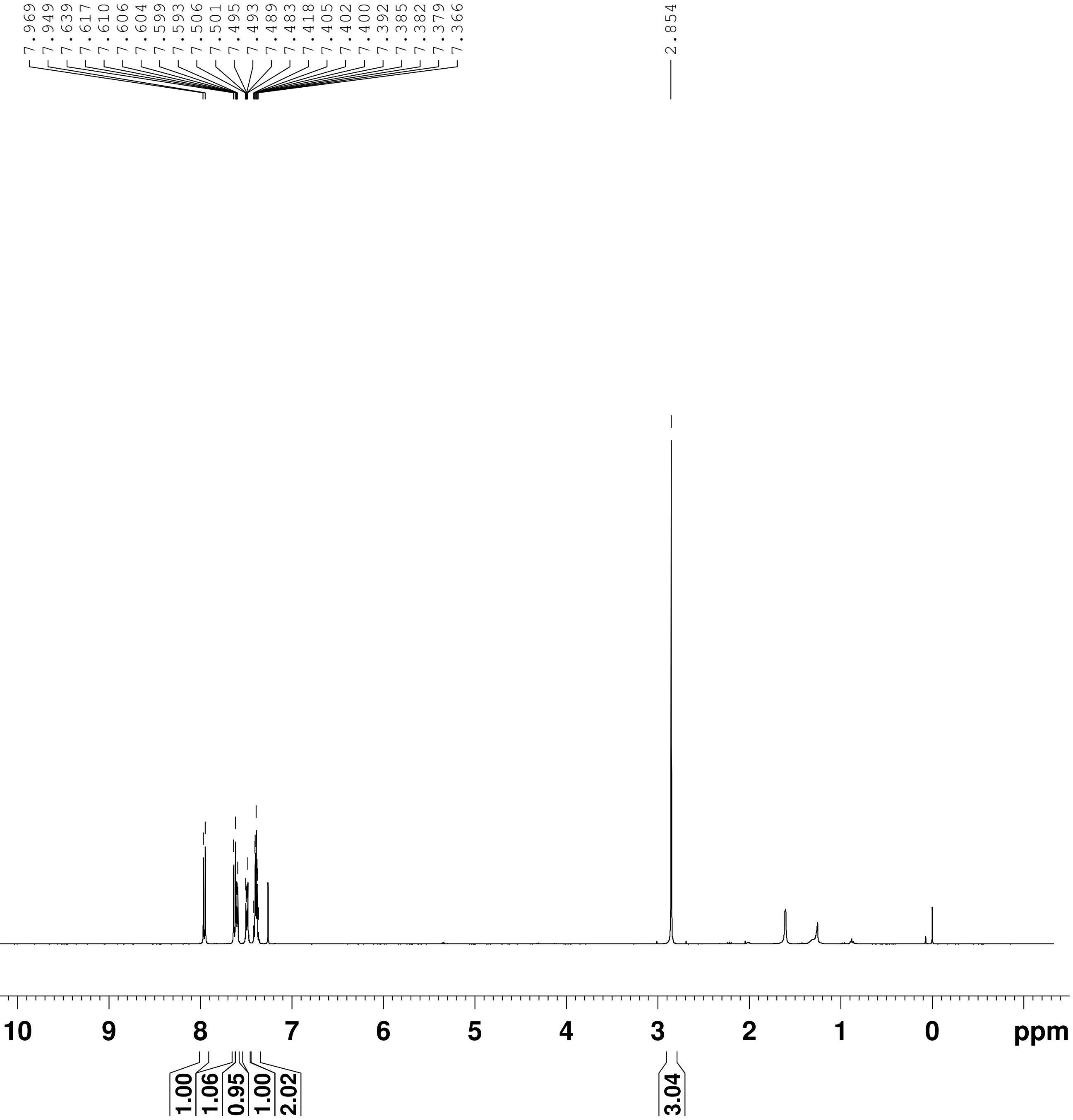

NAME	LV-MM-71-20240807
EXPNO	72
PROCNO	1
Date_	20240807
Time	19.57 h
INSTRUM	Avance
PROBHD	Z163739_0744 (
PULPROG	zg30
TD	65536
SOLVENT	CDCl3
NS	8
DS	0
SWH	6250.000 Hz
FIDRES	0.190735 Hz
AQ	5.2429299 sec
RG	101
DW	80.000 usec
DE	8.64 usec
TE	298.0 K
D1	1.00000000 sec
TD0	1
SFO1	400.1326008 MHz
NUC1	1H
P0	2.67 usec
P1	8.00 usec
SI	65536
SF	400.1300091 MHz
WDW	EM
SSB	0
LB	0.30 Hz
GB	0
PC	1.00

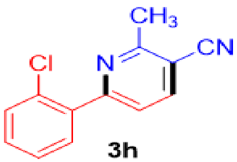

3h

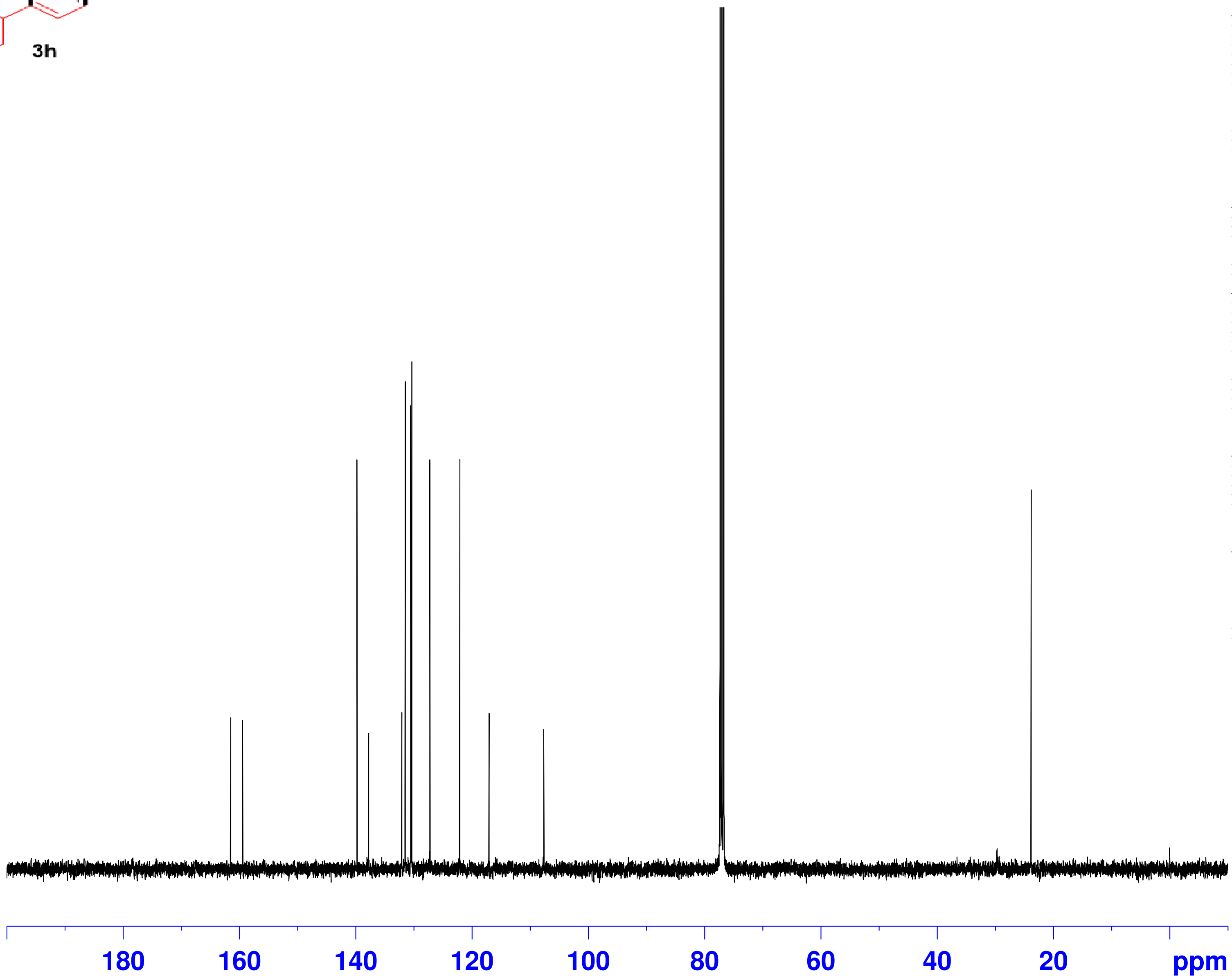

BRUKER

NAME	LV-MM-71-20240807
EXPNO	73
PROCNO	1
Date_	20240807
Time	20.57 h
INSTRUM	Avance
PROBHD	Z163739_0744 (
PULPROG	zgpg30
TD	65536
SOLVENT	CDCl3
NS	1024
DS	4
SWH	23809.523 Hz
FIDRES	0.726609 Hz
AQ	1.3763061 se
RG	101
DW	21.000 us
DE	6.50 us
TE	298.0 K
D1	2.00000000 se
D11	0.03000000 se
TD0	1
SFO1	100.6228298 MH
NUC1	13C
P0	2.67 us
P1	8.00 us
SI	32768
SF	100.6127685 MH
WDW	EM
SSB	0
LB	1.00 Hz
GB	0
PC	1.40

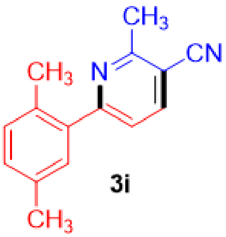

3i

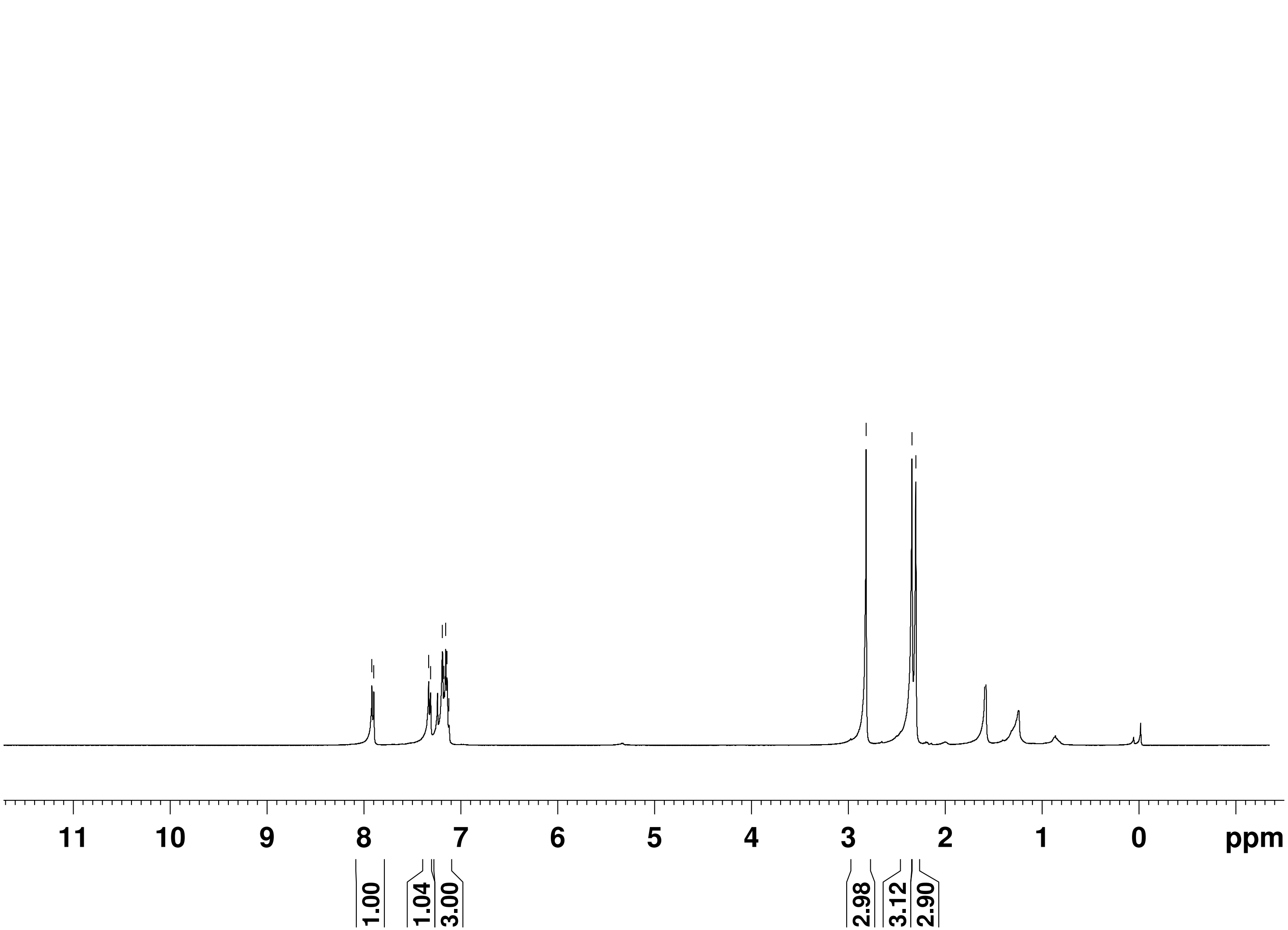

NAME	LV-MM-60-20240806
EXPNO	32
PROCNO	1
Date_	20240807
Time	2.35 h
INSTRUM	Avance
PROBHD	Z163739_0744 (
PULPROG	zg30
TD	65536
SOLVENT	CH2Cl2
NS	8
DS	0
SWH	6250.000 Hz
FIDRES	0.190735 Hz
AQ	5.2429299 sec
RG	101
DW	80.000 usec
DE	8.64 usec
TE	298.0 K
D1	1.00000000 sec
TD0	1
SFO1	400.1326008 MHz
NUC1	1H
P0	2.67 usec
P1	8.00 usec
SI	65536
SF	400.1300175 MHz
WDW	EM
SSB	0
LB	0.30 Hz
GB	0
PC	1.00

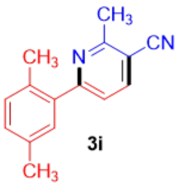

3i

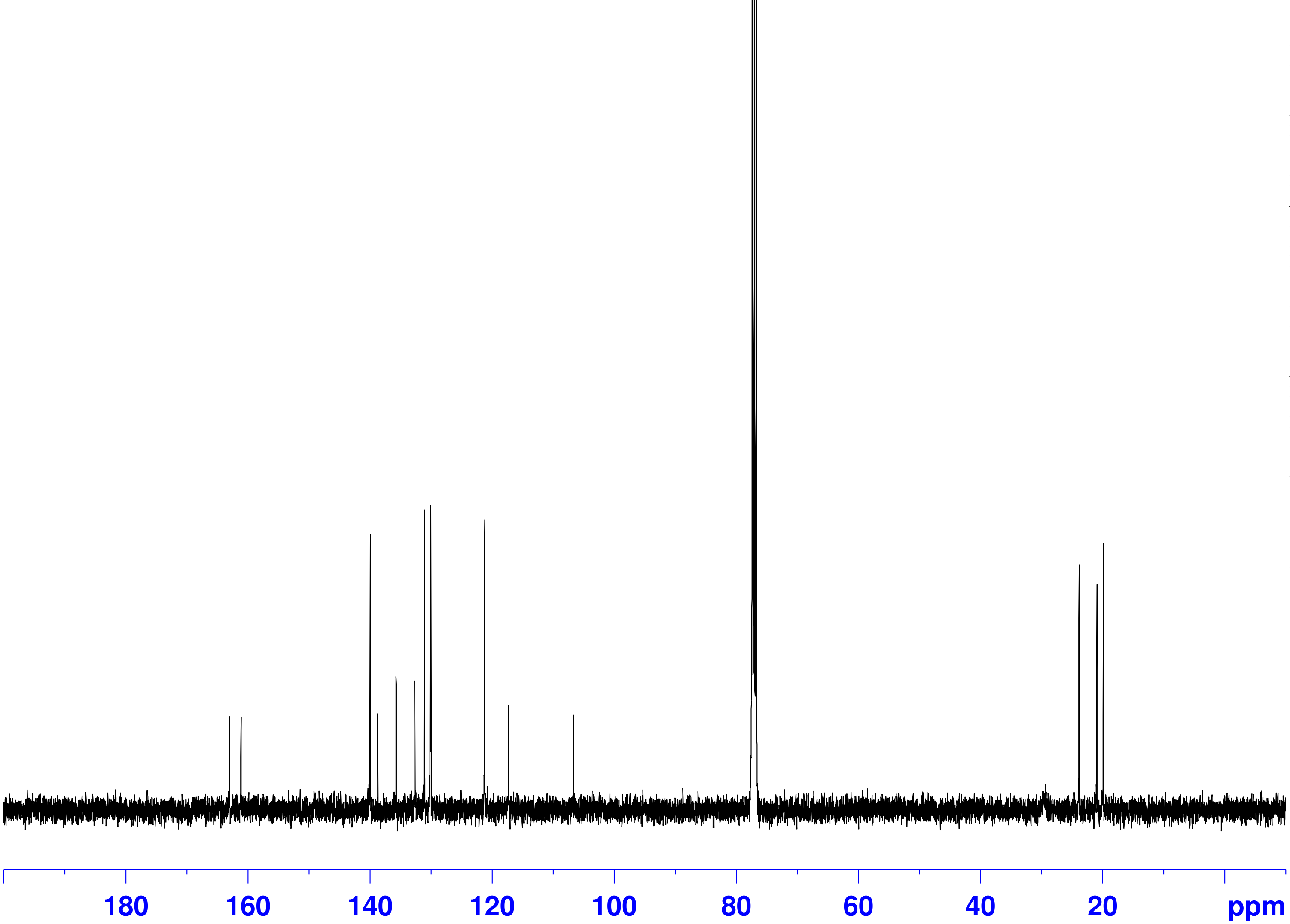

NAME	LV-MM-60-20240806
EXPNO	33
PROCNO	1
Date_	20240807
Time	3.35 h
INSTRUM	Avance
PROBHD	Z163739_0744 (
PULPROG	zgpg30
TD	65536
SOLVENT	CH2Cl2
NS	1024
DS	4
SWH	23809.523 Hz
FIDRES	0.726609 Hz
AQ	1.3763061 se
RG	101
DW	21.000 us
DE	6.50 us
TE	298.0 K
D1	2.00000000 se
D11	0.03000000 se
TD0	1
SFO1	100.6228298 MH
NUC1	13C
P0	2.67 us
P1	8.00 us
SI	32768
SF	100.6127685 MH
WDW	EM
SSB	0
LB	1.00 Hz
GB	0
PC	1.40

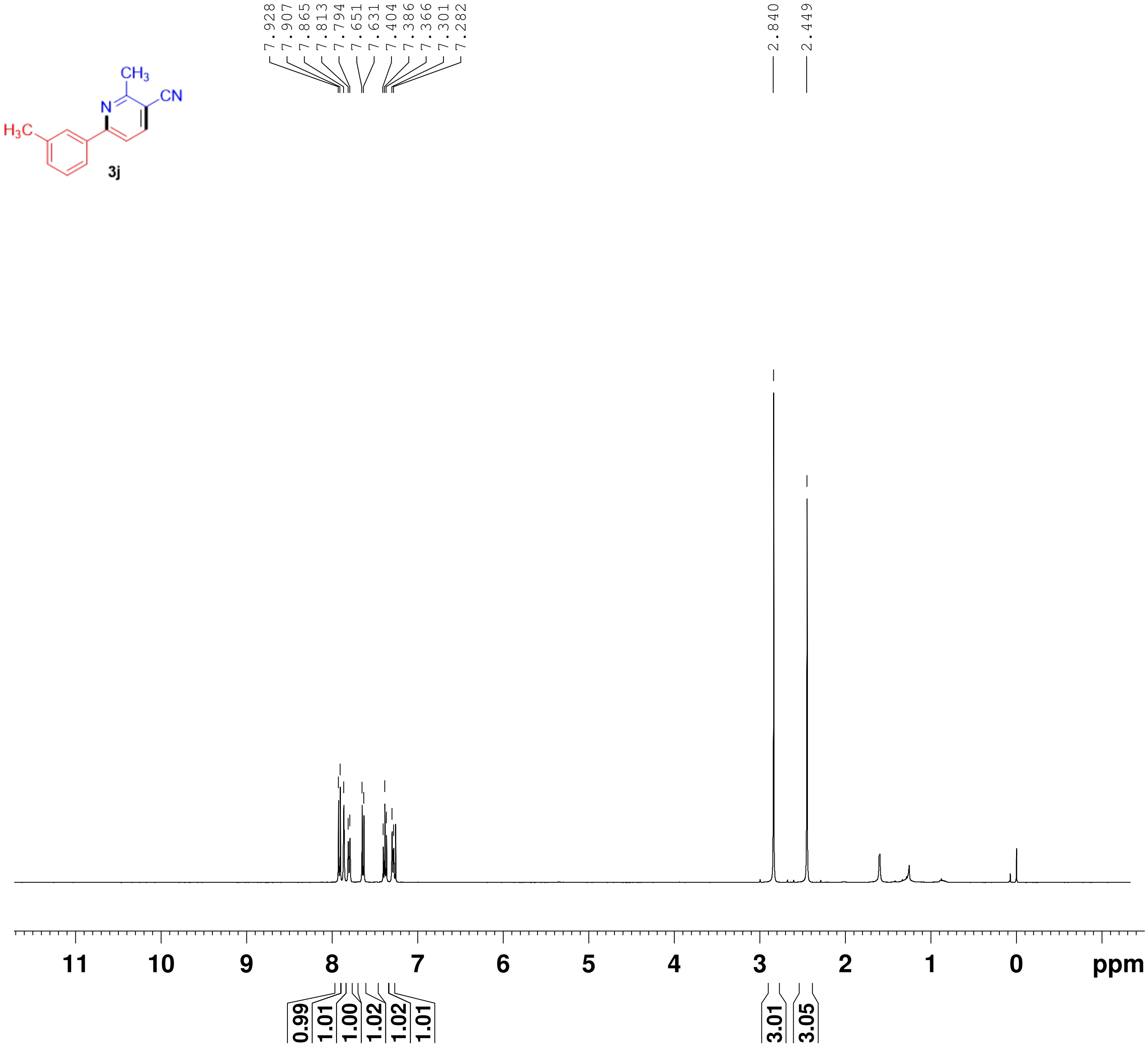

BRUKER

NAME	LV-MM-70-20240807
EXPNO	62
PROCNO	1
Date_	20240807
Time	18.51 h
INSTRUM	Avance
PROBHD	Z163739_0744 (
PULPROG	zg30
TD	65536
SOLVENT	CDCl3
NS	8
DS	0
SWH	6250.000 Hz
FIDRES	0.190735 Hz
AQ	5.2429299 sec
RG	101
DW	80.000 usec
DE	8.64 usec
TE	298.0 K
D1	1.00000000 sec
TD0	1
SFO1	400.1326008 MHz
NUC1	1H
P0	2.67 usec
P1	8.00 usec
SI	65536
SF	400.1300104 MHz
WDW	EM
SSB	0
LB	0.30 Hz
GB	0
PC	1.00

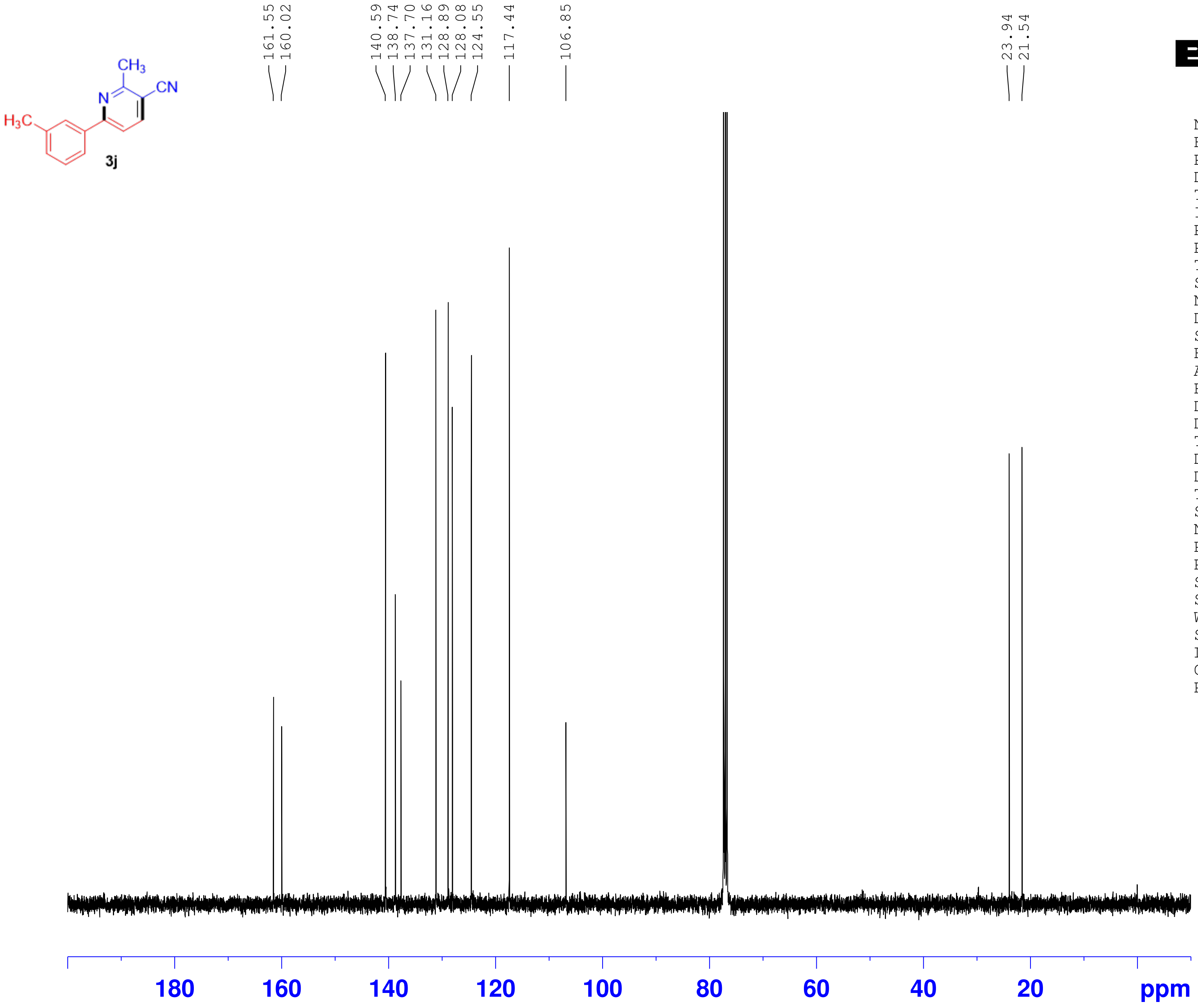

NAME	LV-MM-70-20240807
EXPNO	63
PROCNO	1
Date_	20240807
Time	19.51 h
INSTRUM	Avance
PROBHD	Z163739_0744 (
PULPROG	zgpg30
TD	65536
SOLVENT	CDCl3
NS	1024
DS	4
SWH	23809.523 Hz
FIDRES	0.726609 Hz
AQ	1.3763061 se
RG	101
DW	21.000 us
DE	6.50 us
TE	298.0 K
D1	2.00000000 se
D11	0.03000000 se
TD0	1
SFO1	100.6228298 MH
NUC1	13C
P0	2.67 us
P1	8.00 us
SI	32768
SF	100.6127685 MH
WDW	EM
SSB	0
LB	1.00 Hz
GB	0
PC	1.40

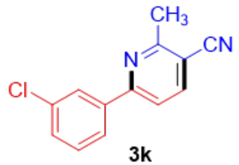

3k

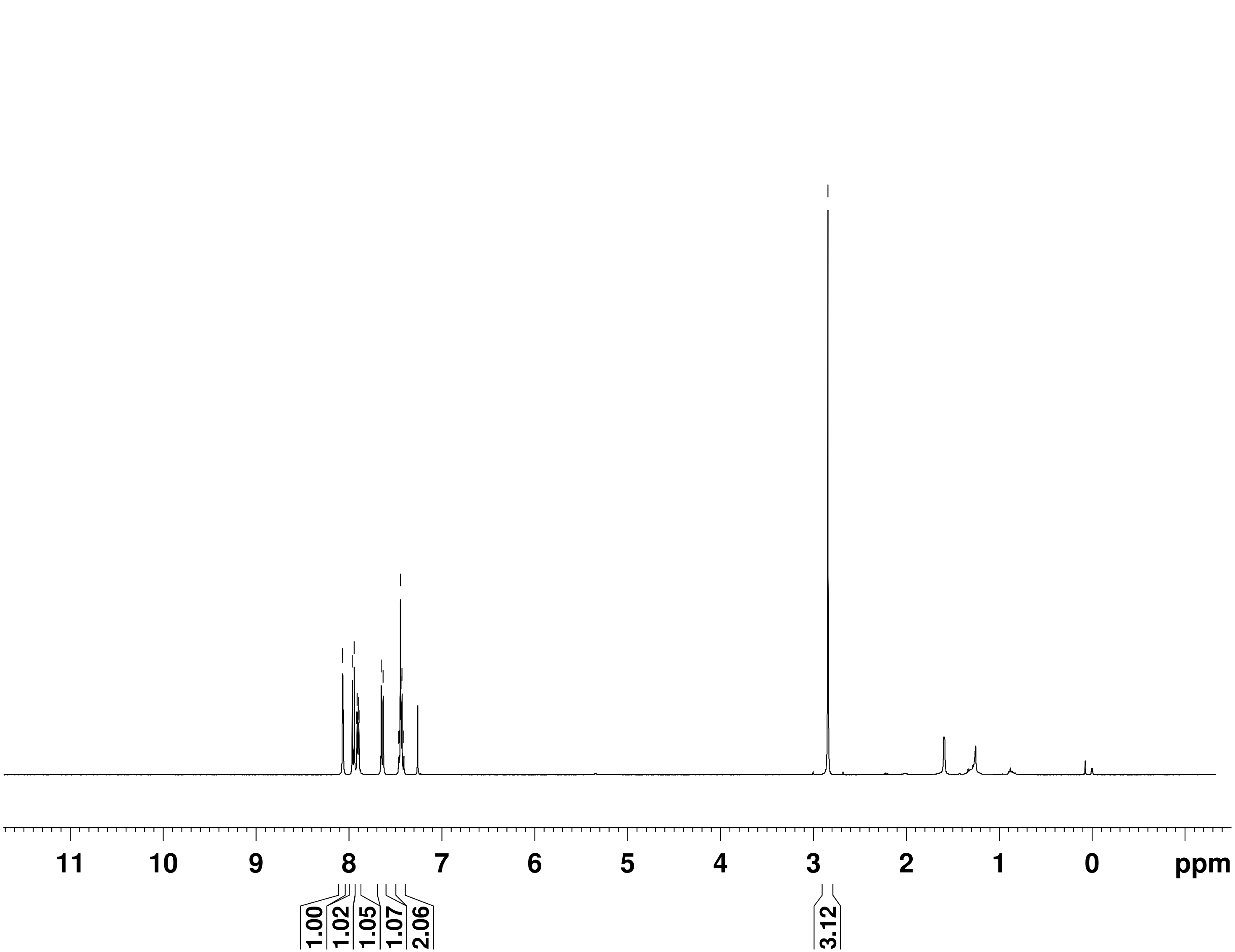

NAME	LV-MM-59-20240806
EXPNO	10
PROCNO	1
Date_	20240807
Time	4.58 h
INSTRUM	Avance
PROBHD	Z163739_0744 (
PULPROG	zg30
TD	65536
SOLVENT	CDCl3
NS	8
DS	0
SWH	6250.000 Hz
FIDRES	0.190735 Hz
AQ	5.2429299 sec
RG	101
DW	80.000 usec
DE	8.64 usec
TE	298.0 K
D1	1.00000000 sec
TD0	1
SFO1	400.1326008 MHz
NUC1	1H
P0	2.67 usec
P1	8.00 usec
SI	65536
SF	400.1300095 MHz
WDW	EM
SSB	0
LB	0.30 Hz
GB	0
PC	1.00

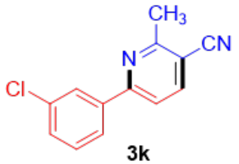

3k

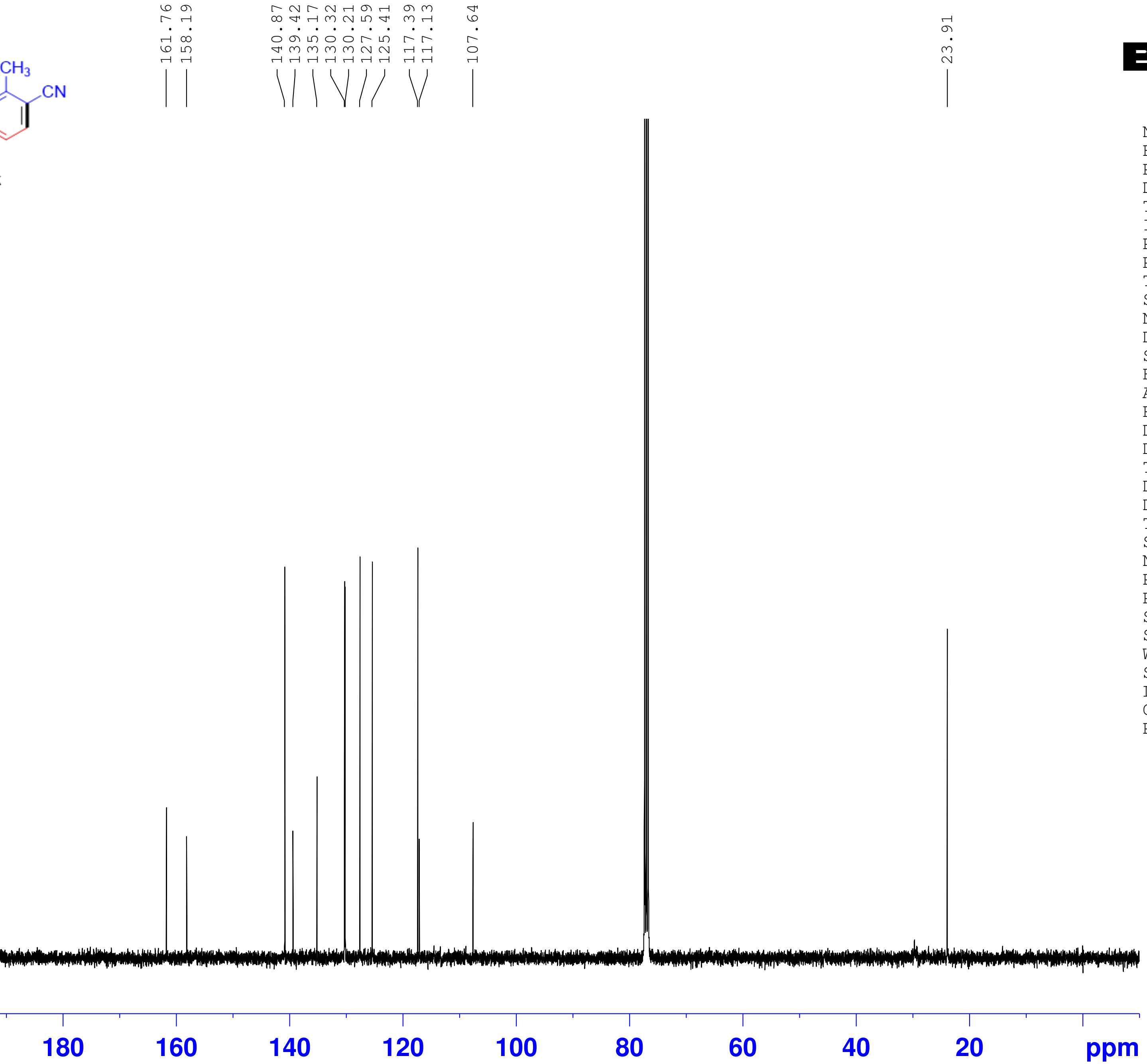

NAME	LV-MM-59-20240806
EXPNO	11
PROCNO	1
Date_	20240807
Time	5.58 h
INSTRUM	Avance
PROBHD	Z163739_0744 (
PULPROG	zgpg30
TD	65536
SOLVENT	CDCl3
NS	1024
DS	4
SWH	23809.523 Hz
FIDRES	0.726609 Hz
AQ	1.3763061 se
RG	101
DW	21.000 us
DE	6.50 us
TE	298.0 K
D1	2.00000000 se
D11	0.03000000 se
TD0	1
SFO1	100.6228298 MH
NUC1	13C
P0	2.67 us
P1	8.00 us
SI	32768
SF	100.6127685 MH
WDW	EM
SSB	0
LB	1.00 Hz
GB	0
PC	1.40

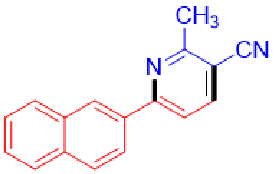

3l

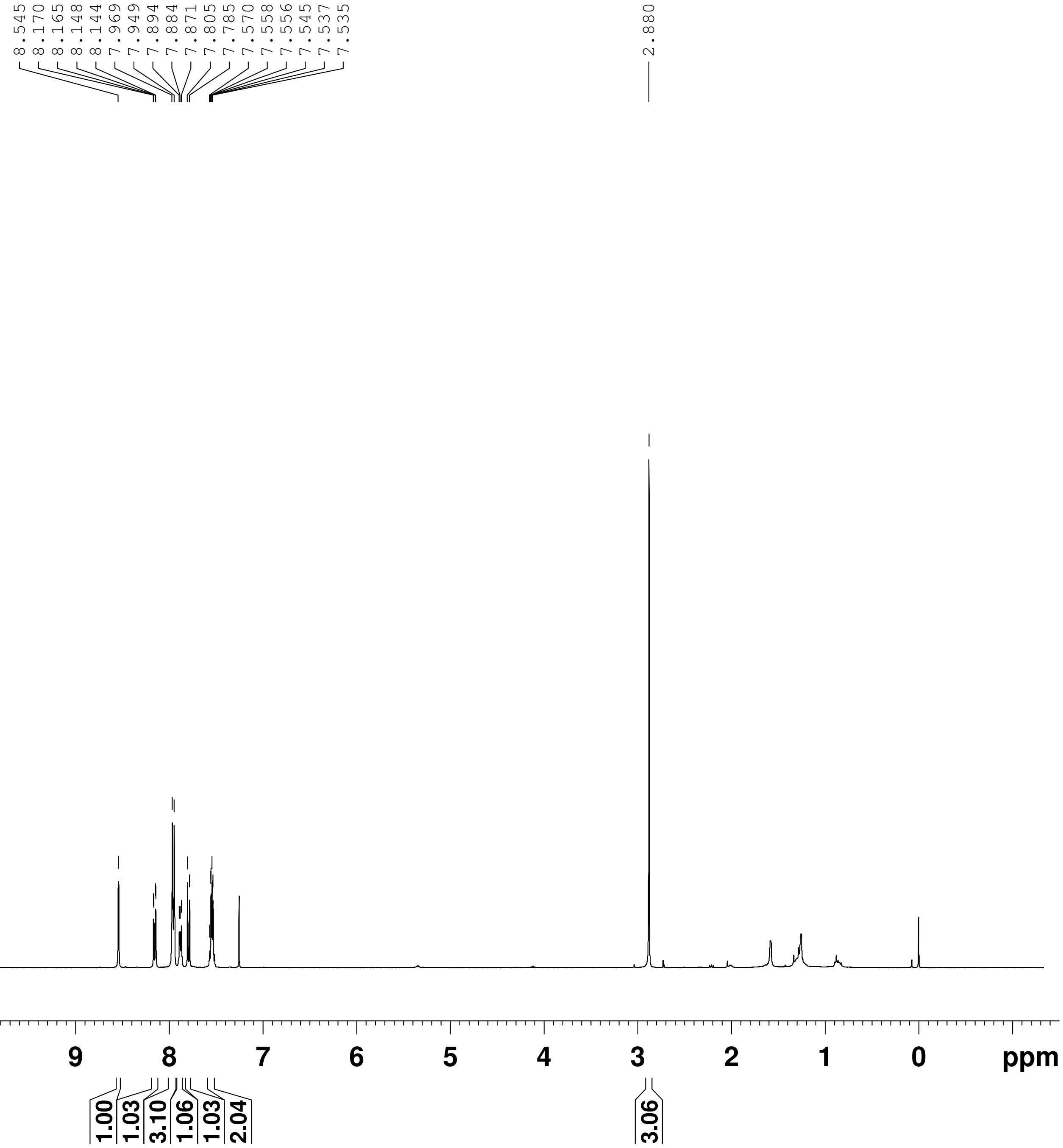

BRUKER

NAME	LV-MM-47-20240804
EXPNO	32
PROCNO	1
Date_	20240805
Time	0.41 h
INSTRUM	Avance
PROBHD	Z163739_0744 (
PULPROG	zg30
TD	65536
SOLVENT	CDCl3
NS	8
DS	0
SWH	6250.000 Hz
FIDRES	0.190735 Hz
AQ	5.2429299 sec
RG	101
DW	80.000 usec
DE	8.64 usec
TE	298.0 K
D1	1.00000000 sec
TD0	1
SFO1	400.1326008 MHz
NUC1	1H
P0	2.67 usec
P1	8.00 usec
SI	65536
SF	400.1300117 MHz
WDW	EM
SSB	0
LB	0.30 Hz
GB	0
PC	1.00

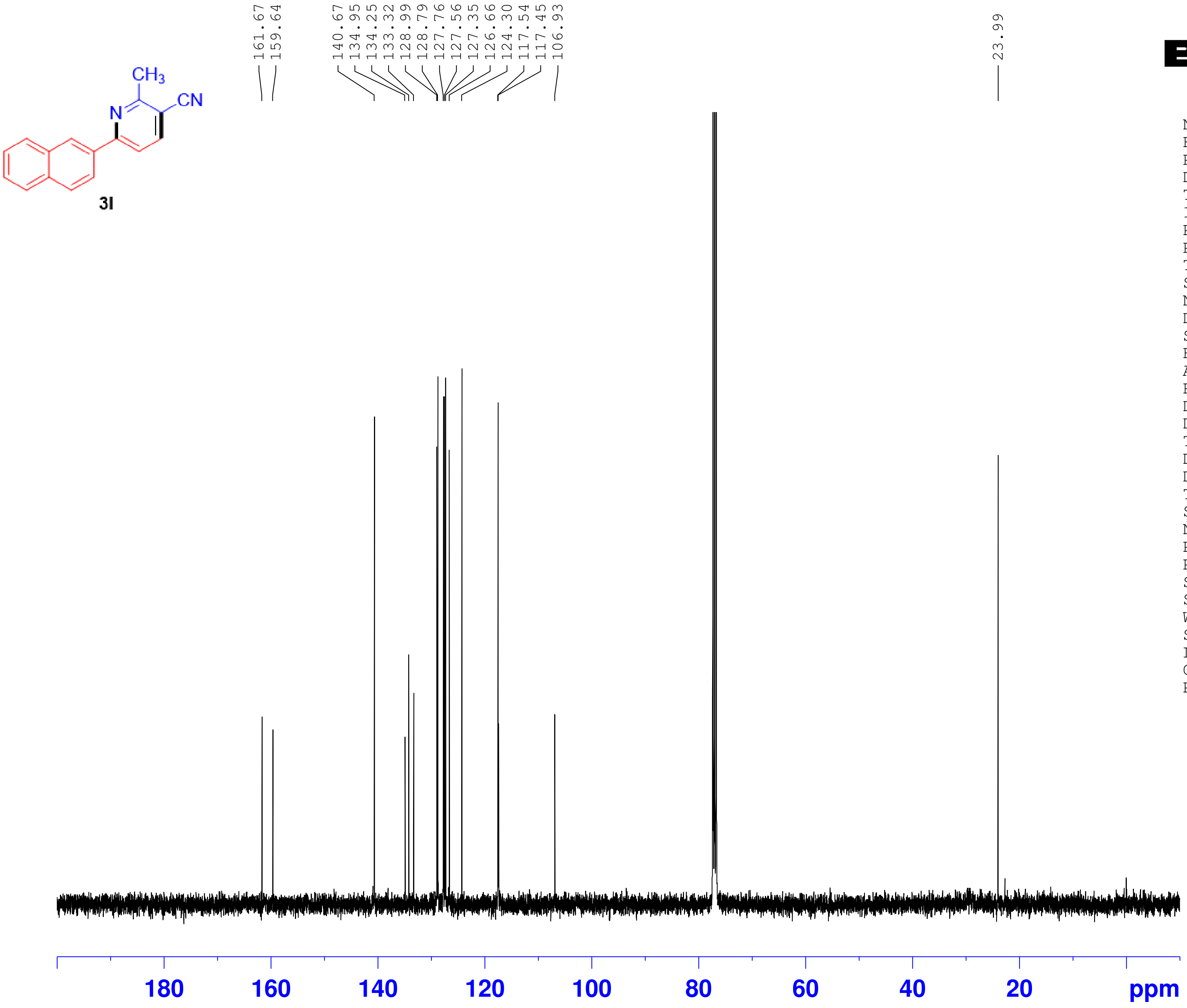

NAME	LV-MM-47-20240804
EXPNO	33
PROCNO	1
Date_	20240805
Time	0.40 h
INSTRUM	Avance
PROBHD	Z163739_0744 (
PULPROG	zgpg30
TD	65536
SOLVENT	CDCl3
NS	1024
DS	4
SWH	23809.523 Hz
FIDRES	0.726609 Hz
AQ	1.3763061 se
RG	101
DW	21.000 us
DE	6.50 us
TE	298.0 K
D1	2.00000000 se
D11	0.03000000 se
TD0	1
SFO1	100.6228298 MH
NUC1	13C
P0	2.67 us
P1	8.00 us
SI	32768
SF	100.6127685 MH
WDW	EM
SSB	0
LB	1.00 Hz
GB	0
PC	1.40

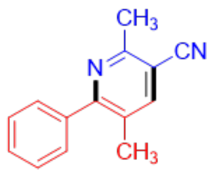

3m

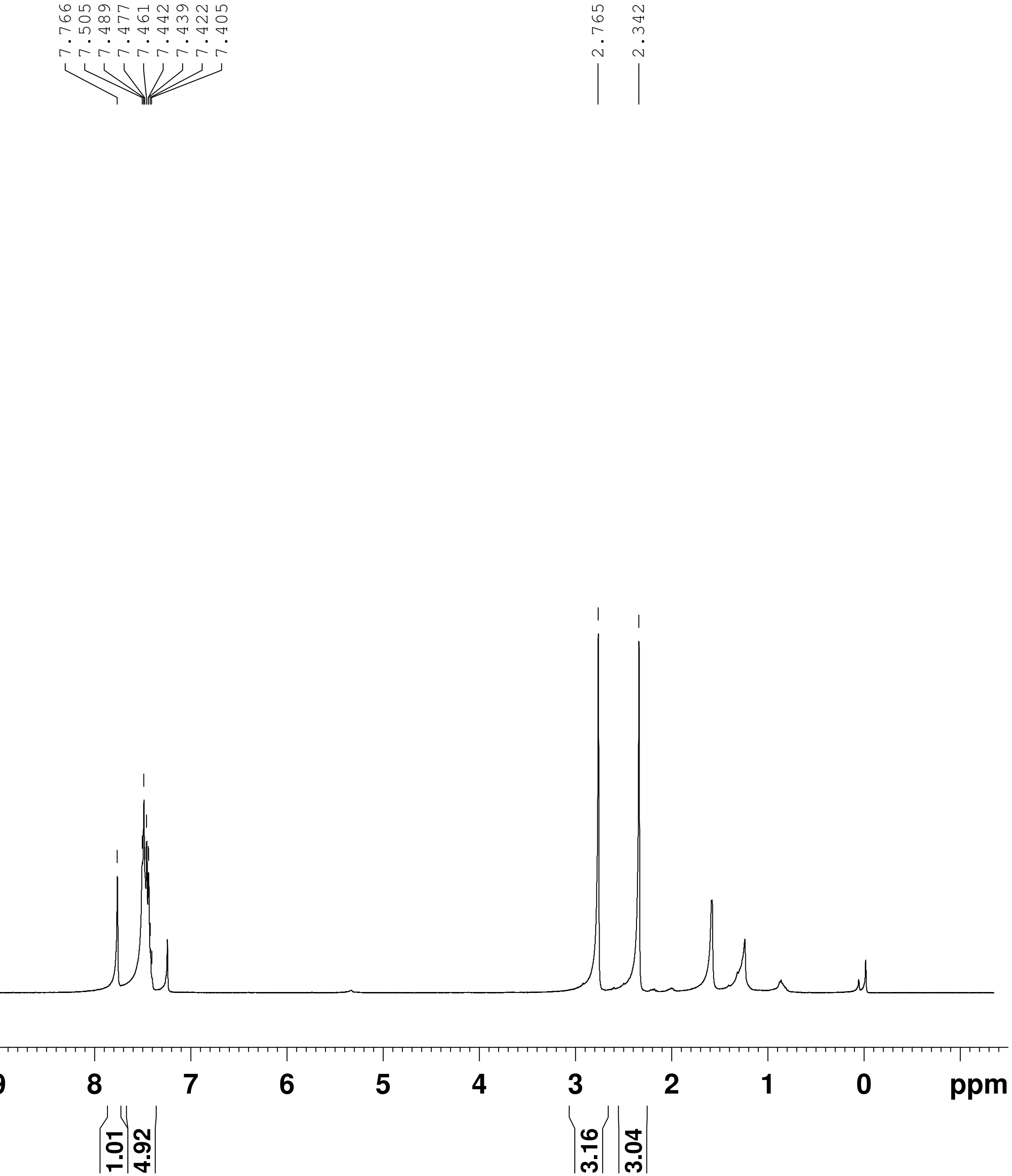

NAME	LV-MM-58-20240806
EXPNO	24
PROCNO	1
Date_	20240807
Time	1.32 h
INSTRUM	Avance
PROBHD	Z163739_0744 (
PULPROG	zg30
TD	65536
SOLVENT	CH2Cl2
NS	8
DS	0
SWH	6250.000 Hz
FIDRES	0.190735 Hz
AQ	5.2429299 sec
RG	101
DW	80.000 usec
DE	8.64 usec
TE	298.0 K
D1	1.00000000 sec
TD0	1
SFO1	400.1326008 MHz
NUC1	1H
P0	2.67 usec
P1	8.00 usec
SI	65536
SF	400.1300162 MHz
WDW	EM
SSB	0
LB	0.30 Hz
GB	0
PC	1.00

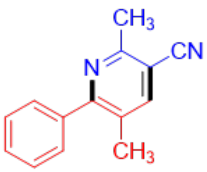

3m

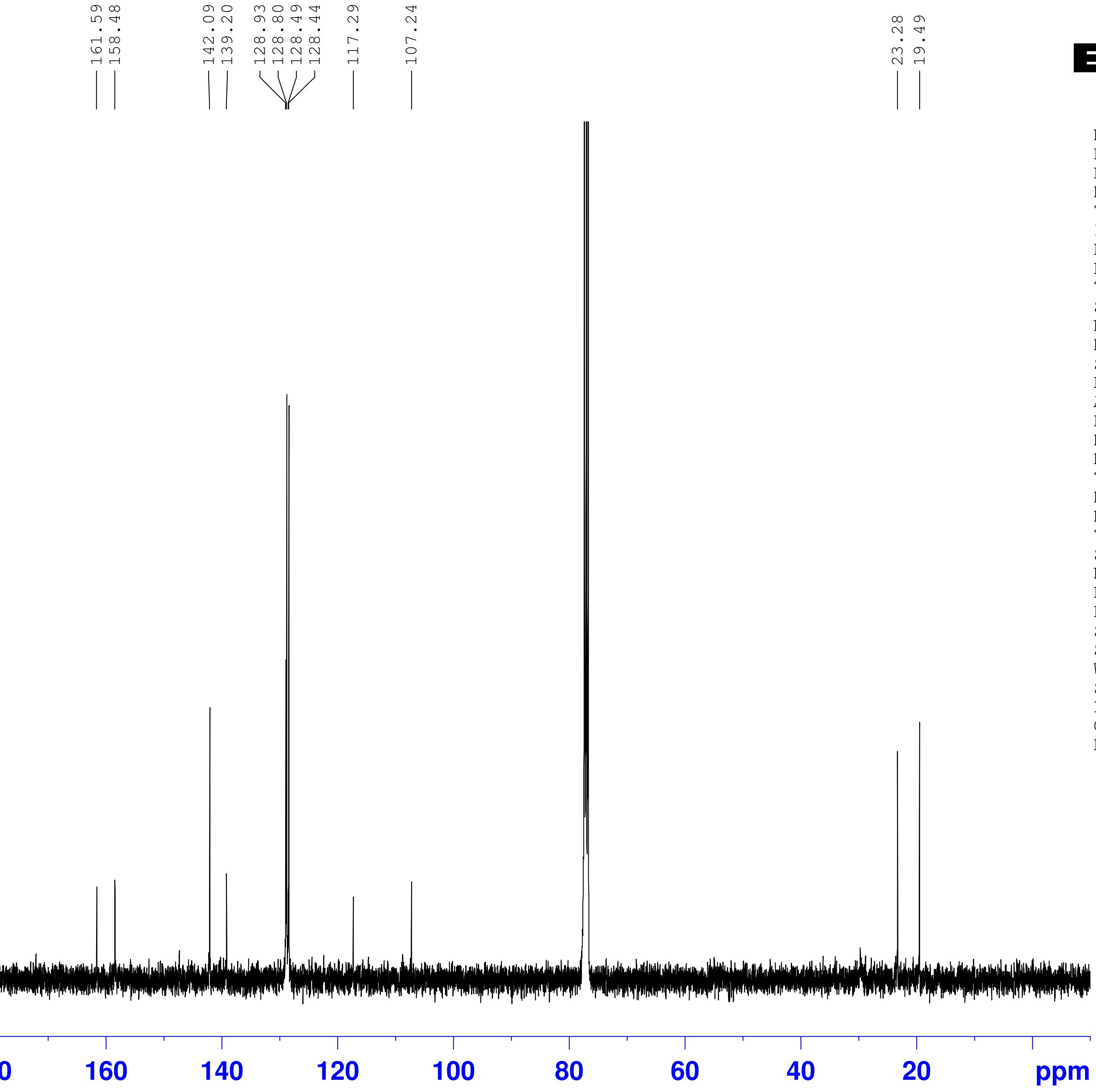

NAME	LV-MM-58-20240806
EXPNO	25
PROCNO	1
Date_	20240807
Time	2.32 h
INSTRUM	Avance
PROBHD	Z163739_0744 (
PULPROG	zgpg30
TD	65536
SOLVENT	CH2Cl2
NS	1024
DS	4
SWH	23809.523 Hz
FIDRES	0.726609 Hz
AQ	1.3763061 se
RG	101
DW	21.000 us
DE	6.50 us
TE	298.0 K
D1	2.00000000 se
D11	0.03000000 se
TD0	1
SFO1	100.6228298 MH
NUC1	13C
P0	2.67 us
P1	8.00 us
SI	32768
SF	100.6127685 MH
WDW	EM
SSB	0
LB	1.00 Hz
GB	0
PC	1.40

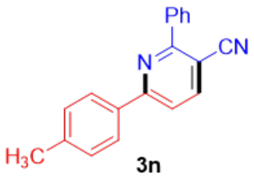

3n

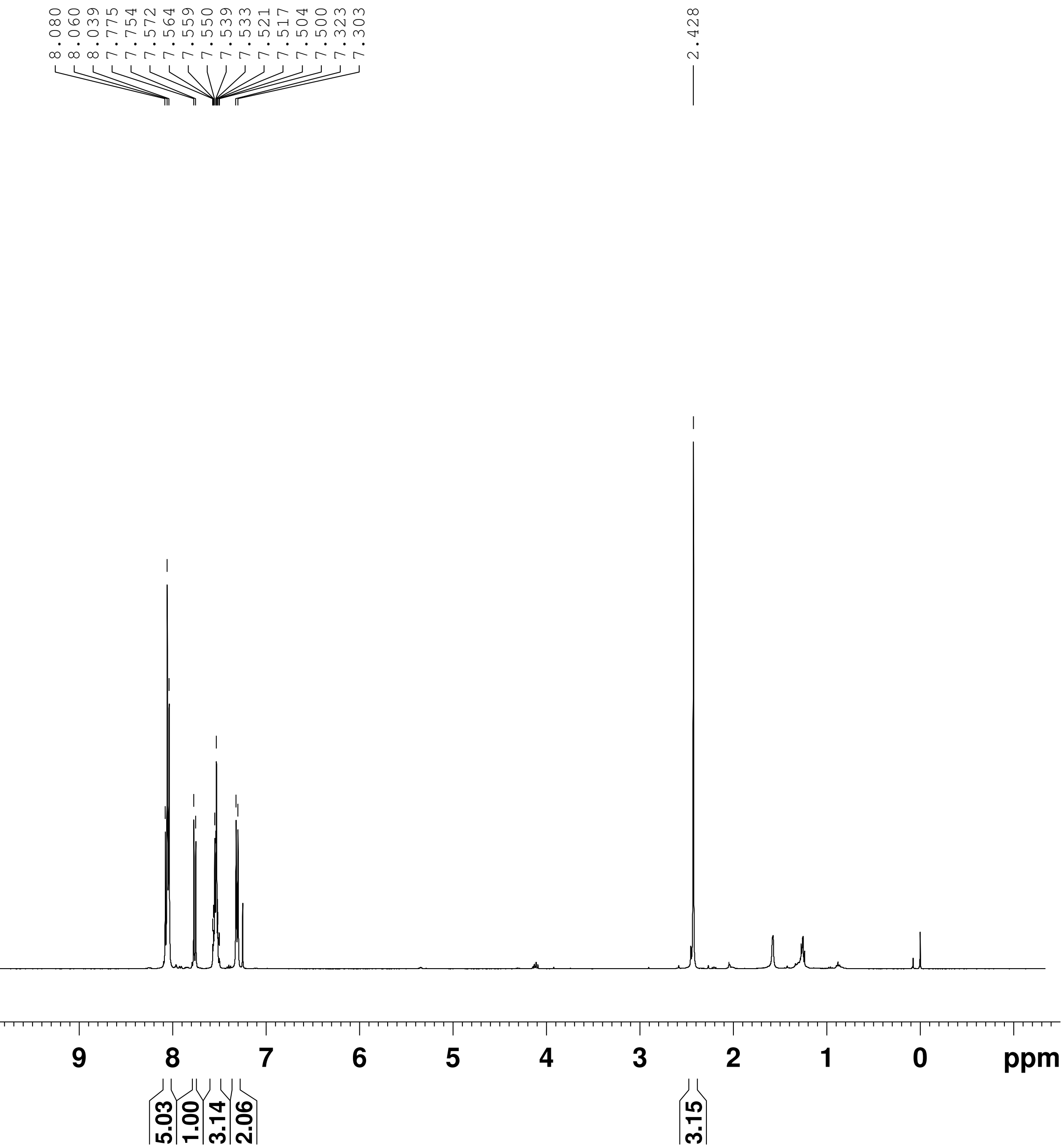

NAME	LV-MM-80-20240808
EXPNO	22
PROCNO	1
Date_	20240809
Time	1.06 h
INSTRUM	Avance
PROBHD	Z163739_0744 (
PULPROG	zg30
TD	65536
SOLVENT	CDCl3
NS	8
DS	0
SWH	6250.000 Hz
FIDRES	0.190735 Hz
AQ	5.2429299 sec
RG	101
DW	80.000 usec
DE	8.64 usec
TE	298.0 K
D1	1.00000000 sec
TD0	1
SFO1	400.1326008 MHz
NUC1	1H
P0	2.67 usec
P1	8.00 usec
SI	65536
SF	400.1300135 MHz
WDW	EM
SSB	0
LB	0.30 Hz
GB	0
PC	1.00

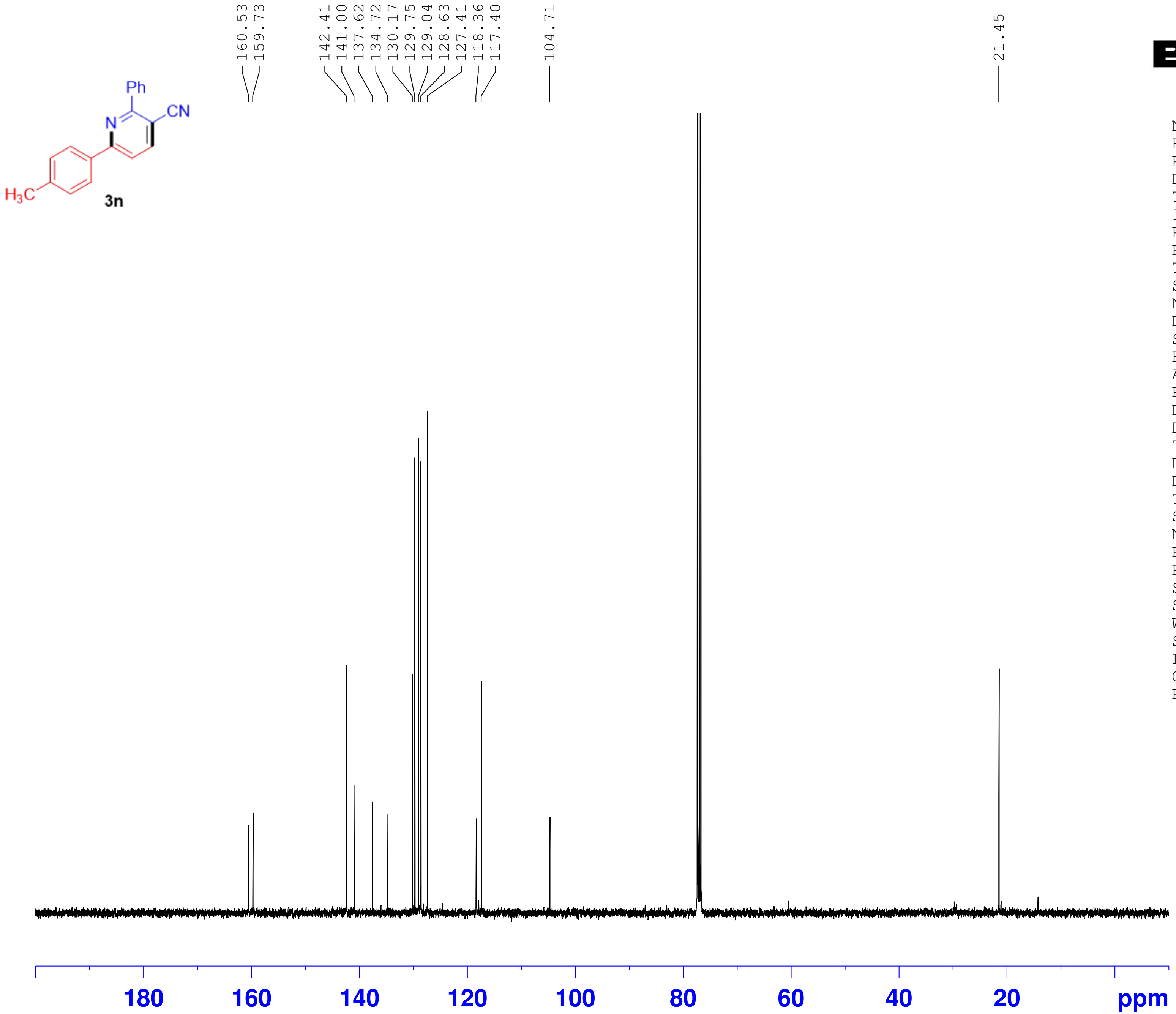

NAME	LV-MM-80-20240808
EXPNO	23
PROCNO	1
Date_	20240809
Time	1.53 h
INSTRUM	Avance
PROBHD	Z163739_0744 (
PULPROG	zgpg30
TD	65536
SOLVENT	CDCl3
NS	800
DS	4
SWH	23809.523 Hz
FIDRES	0.726609 Hz
AQ	1.3763061 se
RG	101
DW	21.000 us
DE	6.50 us
TE	298.0 K
D1	2.00000000 se
D11	0.03000000 se
TD0	1
SFO1	100.6228298 MH
NUC1	13C
P0	2.67 us
P1	8.00 us
SI	32768
SF	100.6127685 MH
WDW	EM
SSB	0
LB	1.00 Hz
GB	0
PC	1.40

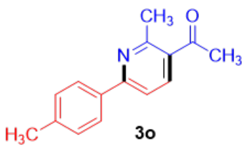

3o

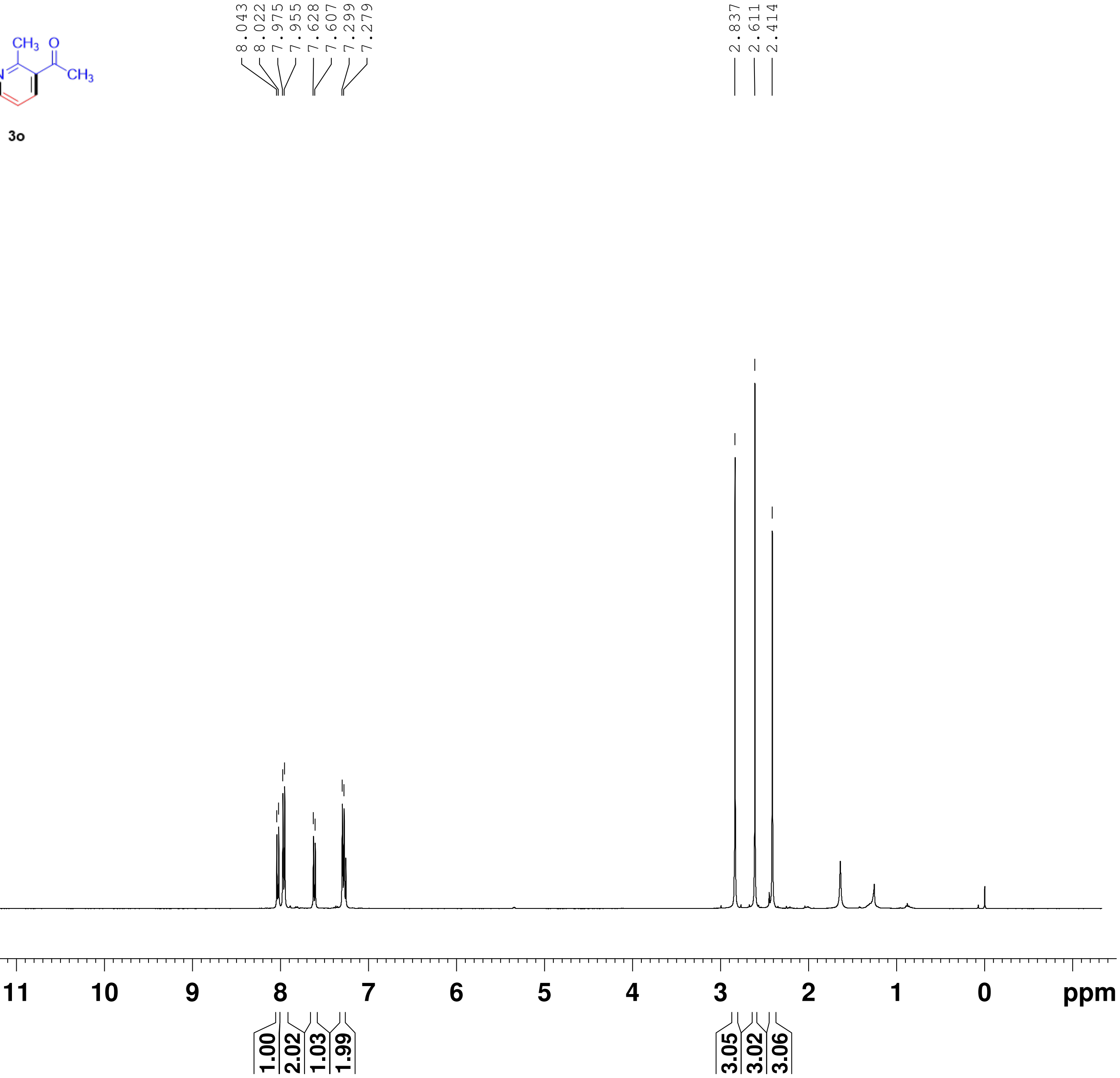

NAME	LV-MM-74-20240810
EXPNO	10
PROCNO	1
Date_	20240810
Time	15.46 h
INSTRUM	Avance
PROBHD	Z163739_0744 (
PULPROG	zg30
TD	65536
SOLVENT	CDCl3
NS	8
DS	0
SWH	6250.000 Hz
FIDRES	0.190735 Hz
AQ	5.2429299 sec
RG	101
DW	80.000 usec
DE	8.64 usec
TE	298.0 K
D1	1.00000000 sec
TD0	1
SFO1	400.1326008 MHz
NUC1	1H
P0	2.67 usec
P1	8.00 usec
SI	65536
SF	400.1300104 MHz
WDW	EM
SSB	0
LB	0.30 Hz
GB	0
PC	1.00

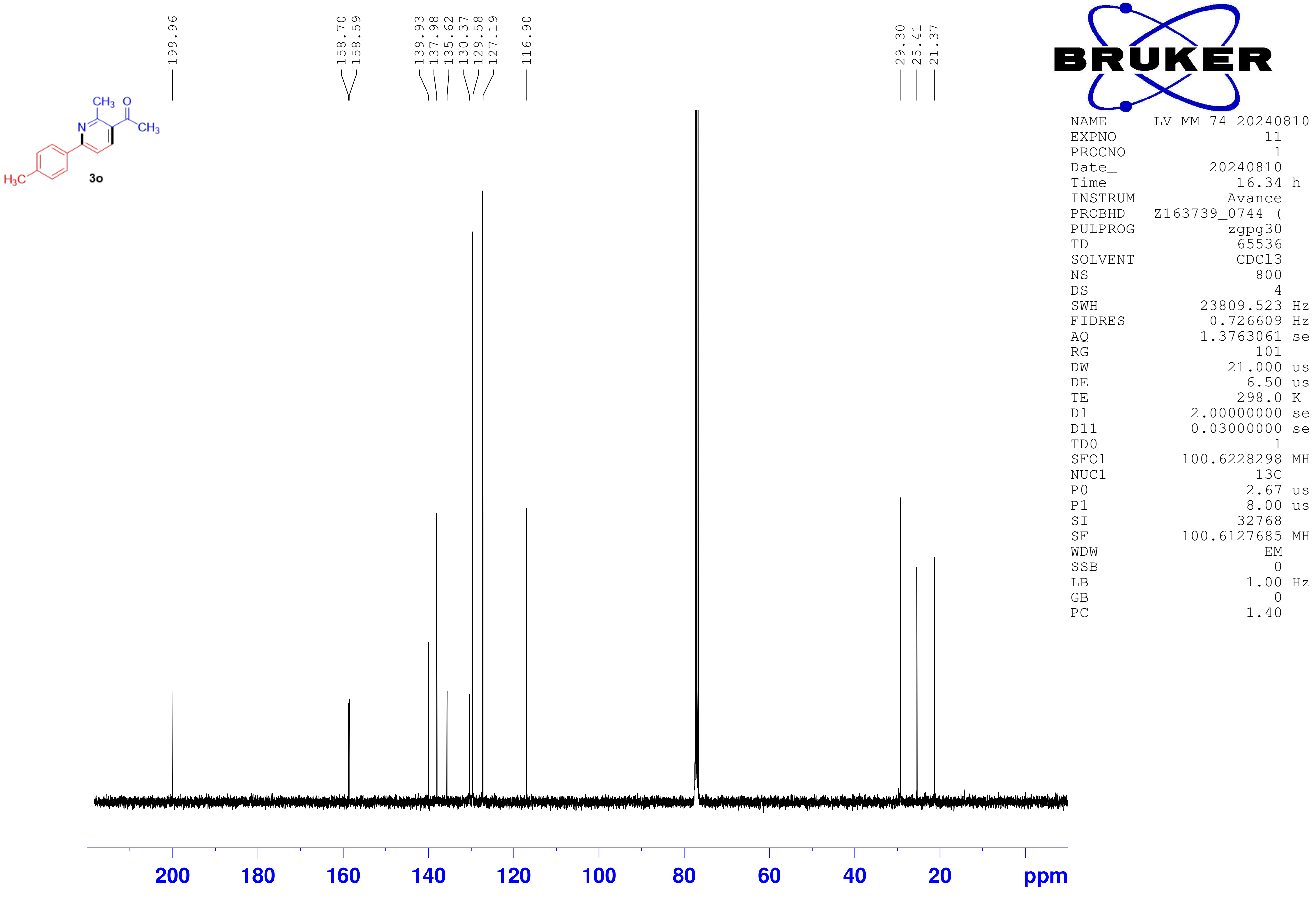
199.96
158.70
158.59
139.93
137.98
135.62
130.37
129.58
127.19
116.90
29.30
25.41
21.37
CH3 O
N
CH3
H3C
3o
BRUKER
NAME LV-MM-74-20240810
EXPNO 11
PROCNO 1
Date_ 20240810
Time 16.34 h
INSTRUM Avance
PROBHD Z163739_0744 (
PULPROG zgpg30
TD 65536
SOLVENT CDCl3
NS 800
DS 4
SWH 23809.523 Hz
FIDRES 0.726609 Hz
AQ 1.3763061 se
RG 101
DW 21.000 us
DE 6.50 us
TE 298.0 K
D1 2.00000000 se
D11 0.03000000 se
TD0 1
SFO1 100.6228298 MH
NUC1 13C
P0 2.67 us
P1 8.00 us
SI 32768
SF 100.6127685 MH
WDW EM
SSB 0
LB 1.00 Hz
GB 0
PC 1.40
200
180
160
140
120
100
80
60
40
20
ppm

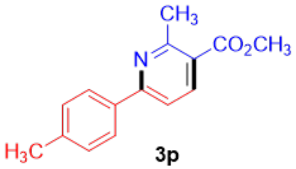

3p

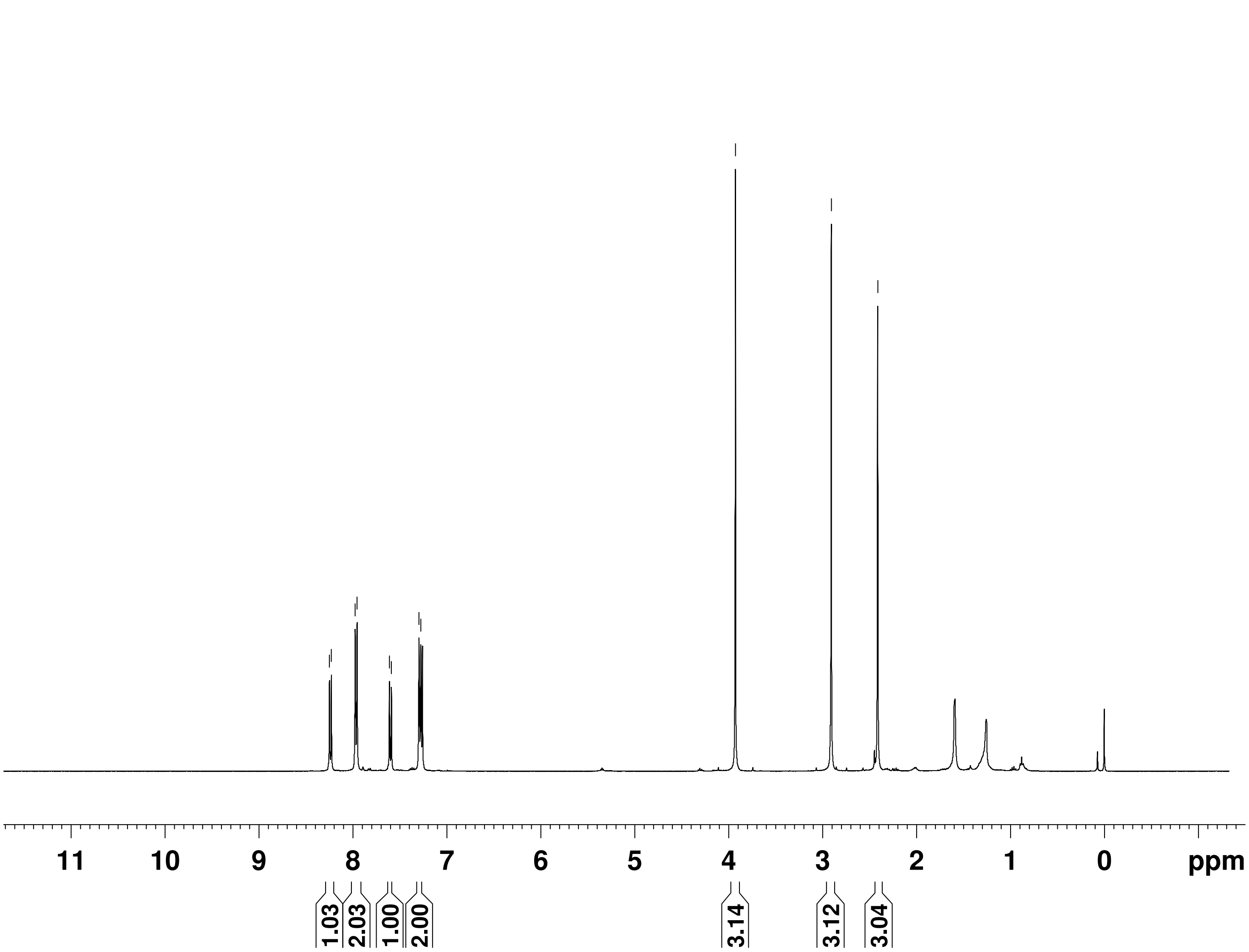

BRUKER

NAME	LV-MM-73-20240808
EXPNO	10
PROCNO	1
Date_	20240809
Time	0.01 h
INSTRUM	Avance
PROBHD	Z163739_0744 (
PULPROG	zg30
TD	65536
SOLVENT	CDCl3
NS	8
DS	0
SWH	6250.000 Hz
FIDRES	0.190735 Hz
AQ	5.2429299 sec
RG	101
DW	80.000 usec
DE	8.64 usec
TE	298.0 K
D1	1.00000000 sec
TD0	1
SFO1	400.1326008 MHz
NUC1	1H
P0	2.67 usec
P1	8.00 usec
SI	65536
SF	400.1300102 MHz
WDW	EM
SSB	0
LB	0.30 Hz
GB	0
PC	1.00

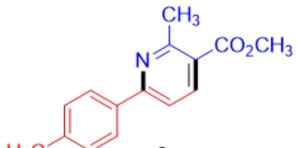

3p

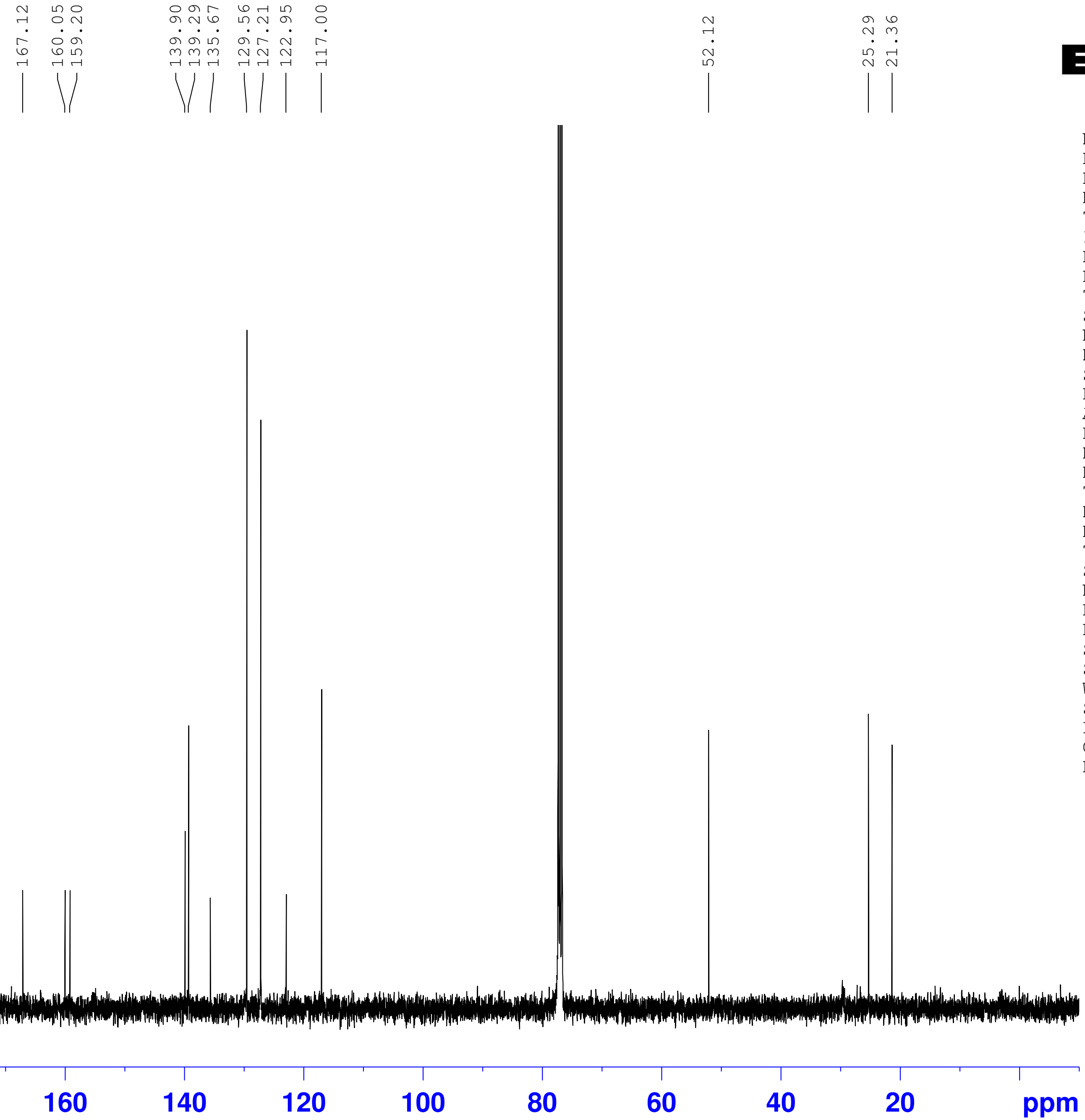

NAME	LV-MM-73-20240808
EXPNO	11
PROCNO	1
Date_	20240809
Time	1.00 h
INSTRUM	Avance
PROBHD	Z163739_0744 (
PULPROG	zgpg30
TD	65536
SOLVENT	CDCl3
NS	1024
DS	4
SWH	23809.523 Hz
FIDRES	0.726609 Hz
AQ	1.3763061 se
RG	101
DW	21.000 us
DE	6.50 us
TE	298.0 K
D1	2.00000000 se
D11	0.03000000 se
TD0	1
SFO1	100.6228298 MH
NUC1	13C
P0	2.67 us
P1	8.00 us
SI	32768
SF	100.6127685 MH
WDW	EM
SSB	0
LB	1.00 Hz
GB	0
PC	1.40
